# Sampling a gradient of red snow algae bloom density reveals novel connections between microbial communities and environmental features

**DOI:** 10.1038/s41598-022-13914-7

**Published:** 2022-06-22

**Authors:** Avery E. Tucker, Shawn P. Brown

**Affiliations:** 1 Department of Biological Sciences, The University of Memphis, Memphis, TN 38152 USA; 2 Center for Biodiversity Research, The University of Memphis, Memphis, TN 38152 USA

**Keywords:** Microbial ecology, Metagenomics

## Abstract

Snow algae blooms and associated microbial communities play large roles in snow ecosystem processes. Patterns and mechanisms underpinning snow algae bloom spatial distribution and associated microbial community assembly dynamics are poorly understood. Here we examine associations of microbial communities and environmental measures between/within snow algae blooms. Snows from the Cascade Mountains and the Rocky Mountains (USA) were collected from medial (M), peripheral (P), and adjacent (A) zones of red snow algae blooms. Medial snow shows increased levels of pollen, lower oxidation–reduction potential, decreased algal and increased bacterial richness, and increased levels of potassium when compared to A and P within the same bloom. Between the Cascade and Rocky Mountains, fungal communities are distinct but bacterial and algal communities show little differentiation. A weighted OTU co-expression analysis (WOCNA) explores OTU modules and their differential correlation with environmental features, suggesting certain subcommunities may be altered by ecological patterns. Individual OTU interaction networks (fungi and bacteria) show high levels of connectivity compared to networks based on the red snow alga *Sanguina nivaloides*, which underscores associative differences between algal dominated networks and other taxa.

## Introduction

Snow algae can form colorful blooms on the snow surface during late-season snow melt. Perhaps the most well-known snow alga is *Sanguina nivaloides* (previously identified as *Chlamydomonas nivalis*)^1^, which forms striking red blooms near the end of the growing season as the UV-protectant astaxanthin and fatty acid ester derivatives accumulate^2^. While *Sanguina* life cycles are unresolved, leading hypotheses include a motile haploid vegetative cell stage that is active during spring or summer snow melt as dissolved nutrients and gases become accessible^3,4^ followed by red cyst formation toward the end of the growing season. Red snow algae blooms also have been a historical curiosity for centuries^5–8^ and more recently are the focus of examinations on algal biodiversity^1,9^ and biogeochemistry ^10–13^, among others. They also contain diverse psychrophilic and psychrotolerant microbes including fungi, bacteria, and archaea^14–17^. While red snow algae blooms are composed of diverse microorganisms there is currently a poor understanding of drivers of community assembly dynamics within these snows.

*S. nivaloides* is found globally in polar and alpine environments where late-season snow persists^1,17–20^; over the course of the last century, they have been widely documented in western North America and these blooms are most often found in open snow fields above timberline^9^. Snow algae blooms are not restricted to *Sanguina* species but are taxonomically diverse and presumably consist of clonal haplotypes of several algal species^18^. Other algal genera often observed in North American red snow algae blooms include *Carteria*, *Chloromonas*, *Chodatella, Cryocystis*, *Raphidonema* and *Trebouxia*, among others^11,21–24^. While not visually apparent, ‘white’ snow with no apparent algal colonization have been demonstrated to contain a multitude of diverse algae, including *Sanguina* spp. as well as diverse and active fungi and bacteria^15,16^.

Microscopy, culture, and metabarcoding-based surveys indicate that fungi and bacteria are common and important components of snow communities associated with snow algae blooms^15,19,23,25–27^. In the Cascade and Rocky Mountains of the United States, diverse fungi belonging to Chytridiomycetes, Dothideomycetes, Eurotiomycetes, Microbotryomycetes, Monoblepharidomycetes, among others are commonly found^15,16,27^. This includes unique clades of snow chytrids found across the Western United States that may or may not parasitize snow algae, but whose full taxonomies and functions are unresolved^15,28^. *S. nivaloides* has also been observed to be closely associated with some gram-negative bacteria^29^, but studies of symbioses are difficult as *S*. *nivaloides* has yet resisted culturing efforts. Sequencing efforts are also challenged by the fact that *S. nivaloides* is commonly observed with its culturable conspecific *Sanguina aurantia*^30^. However, symbiotic effects between other algae and bacteria have been well documented from cultures^31^ and from co-culturing experiments demonstrating enhanced algal growth in the presence of snow bacteria isolates^32,33^.

Snow algae and associated microbial communities are documented to affect biogeochemical cycles through weathering^13^. Recent remote sensing studies underscore the importance of snow algae as a carbon sink, although sparse respiration rate information may indicate only ephemeral sequestration^34^. Further, snow algae blooms have bioalbedo feedback effects that increase snow melt rates^19,35^. While some studies have examined microbial community composition of red snow algae blooms, it is less understood what temporal patterns and environmental features drive community assembly; while it is broadly understood that snow is subject to punctuated and continuous trophic shifts due to dry aeolian deposition, precipitation, and primary productivity by endemic taxa^17^.

Red snow algae blooms typically occur from one to several weeks during spring and summer when air temperatures remain above 0 °C in semi-permanent or perennial snowfields^36^. Estimates of nutrient release from recent snow suggests the first 30% of meltwater contains *ca.* 50–80% of the nutrients^37^, suggesting that endogenous nutrient availability peaks early in the season as snow begins to ablate. Nutrient release may also be facilitated by saprotrophs that breakdown pollen, algae, and other biologic material or dissolved organic matter^16,28^, however, it is not known if this nutrient release is concurrent with, or preceding bloom development. In some cases, snow algae growth can be induced in situ by applications of fertilizer^35^ and, for certain taxa like Chlainomonas sp., growth is thought to be inhibited under acidic conditions, such as those induced by prevailing winds from volcanic eruptions^38^ which likely have strong impacts on snow with limited pH buffering capability^39^.

A study of alpine snow algae in the Sierra Nevada Mountain range of California^40^ and two in the Pacific Northwest^13^ concluded that dissolved inorganic carbon (DIC) is a limiting factor for snow algae primary productivity. Bioavailable P and N did not stimulate bloom growth, but DIC did, while elevated ratios of Fe, Mn and P compared to the local geology were observed, suggesting active sequestration of local weathered rock minerals and/or utilization of diverse substrates. Additional work indicated that inorganic carbon can stimulate snow algae primary productivity^41^.

To address questions of spatial and environmental dynamics on snow algae and associated microbes, blooms found in the Rocky Mountains (Colorado and Wyoming, USA) and the Cascade Mountains (Washington, USA) were demarcated into three visually discernable zones corresponding to algal density and communities were queried using metabarcoding: medial (M) high density; peripheral (P) moderate density; and adjacent (A) no visible snow algae but near to algae blooms^42^. This design was used as sampling zones are easily visualized in the field and is indicative of differing concentrations of snow algae cysts. This research addresses unresolved questions on how microbial communities and environmental features are associated across zones of varying snow algae bloom density. Additional questions ask (1) how the microbial community is structured regionally and/or within individual blooms? (2) What changes in snow physiochemistry accompany algal bloom density? And (3) what co-associations exist between taxa and does this suggest the existence of a core snow algae bloom microbiome? We attempt to answer these questions using standard diversity measures, inferential statistics, and a weighted-OTU correlation network analysis (WOCNA); where the WOCNA identifies correlations between sub-community modules and environmental parameters or parametrized OTUs.

## Results

### Composition and regional differences of snow algae blooms

**Figure 1:**
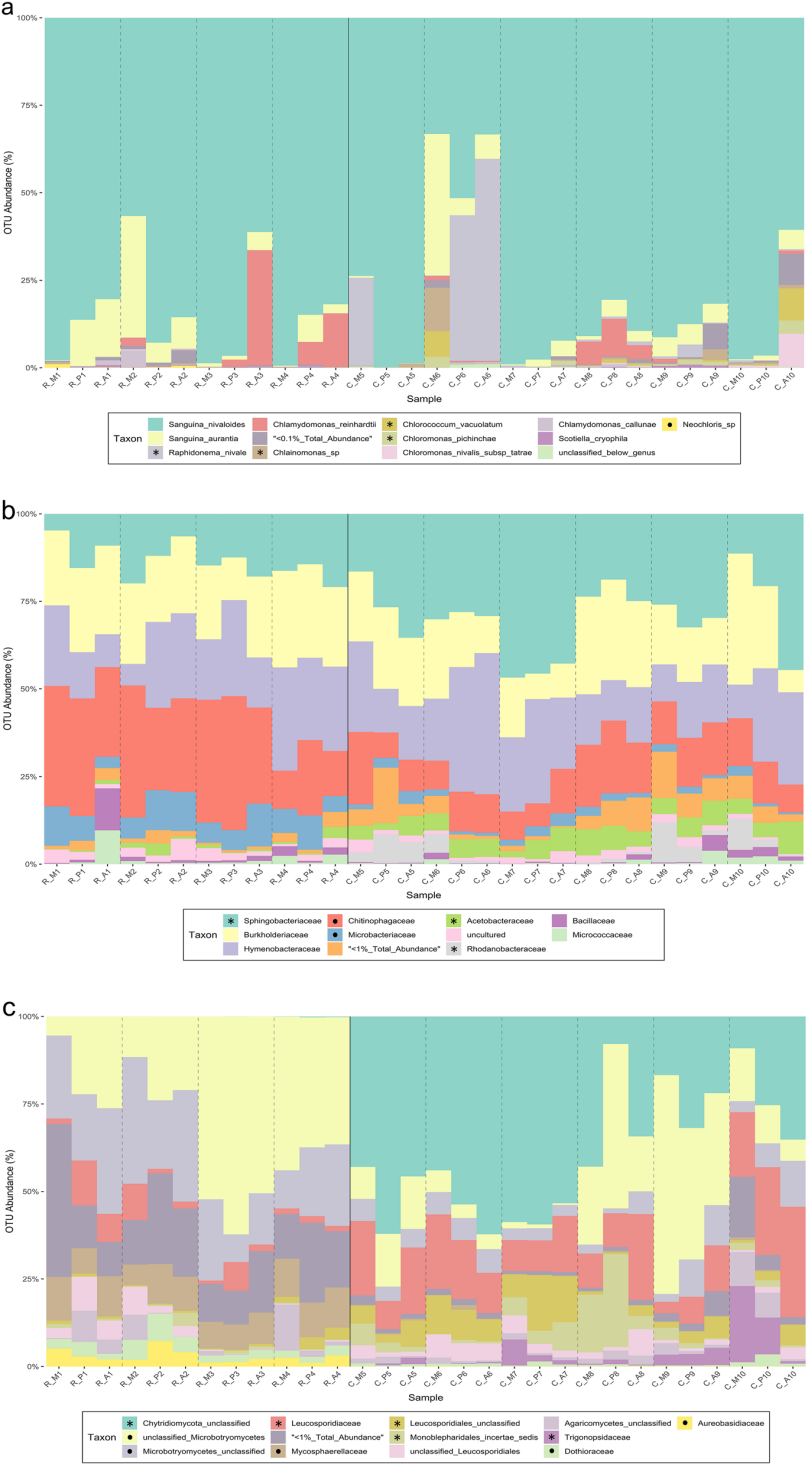
(**a–c**) Algal (**a**), bacterial (**b**), and fungal (**c**) relative abundances resolved at the family, genus, and species levels respectively. Samples from the Cascades (C), Rockies (R) and snow bloom zones (M, P, or A) are noted on the x-axis. Taxa below 0.1% of the total OTU abundance were grouped together. Legend asterisks (*) and circles (⦁) denote taxa with OTU counts found to be significantly higher between the Cascades or Rockies samples respectively (Wilcoxon Rank Sum; p < 0.05). Differences for “< 0.1% Total Abundance” were not tested for significance.

Algal OTU relative abundances (RA%) in the Cascade and Rocky Mountains (Fig. 1a) which were dominated by *Sanguina nivaloides* (86% RA), and less so by *Sanguina aurantia* (5% RA). However, statistically significant median differences (MD) indicate *Raphidonema nivale* (4.5% RA) is more abundant in the Cascades (MD = − 0.006; p = 0.00025) than the Rocky Mountains; positive MD values indicate increased abundance in the Rocky Mountains and negative values indicate increased abundance in the Cascade Mountains.

Bacterial communities were dominated by four families (Fig. 1b): Sphingobacteriaceae, which was more abundant in the Cascades (23% RA; MD = − 0.14; p = 1.10 × 10^–04^), Burkholderiaceae (20% RA), Hymenobacteraceae (20% RA), and Chitinophagaceae, which were more abundant in the Rocky Mountains (19% RA; MD = 0.15; p = 2.60 × 10^–04^). Less dominant taxa (between 1 and 10% RA) included: Microbacteriaceae (4.4% RA; MD = 0.05; p = 3.00 × 10^–06^, Acetobacteraceae (3.5% RA; MD = − 0.05; p = 6.50 × 10^–06^, uncultured (2.1% RA), Rhodanobacteraceae (1.9% RA; MD = − 0.01; p = 1.00 × 10^–04^), Bacillaceae (1.23% RA), and Micrococcaceae (1% RA).

Fungi had higher OTU richness in the Rocky Mountains than the Cascade Mountains (S = 25; p = 0.026), likely due to numerous low-abundant OTUs (< 1% RA) found in the Rockies (Fig. 1c). Unclassified OTUs within the phylum Chytridiomycota (three OTUs), were dominant in the Cascades (38% RA) but nearly absent in the Rockies (0.04% RA). Microbotryomycetes were abundant within both regions, but significantly more so in the Rockies for an unclassified Microbotryomycete OTU (MD = 0.18; p = 0.02) and unclassified families within Microbotryomycetes (MD = 0.16; p = 3.47 × 10^–06^). These two unclassified lineages within Microbotryomycetes were abundant across all samples (40% RA). Other dominant families including Aureobasidiaceae (MD = 0.02; p = 3.30 × 10^–07^), Dothioraceae (MD = 0.02; p = 9.20 × 10^–06^), and Mycosphaerellaceae (MD = 0.10; p = 3.30 × 10^–07^), were more abundant in the Rockies. Leucosporidiaceae (MD = − 0.1; p = 1.30 × 10^–04^), Leucosporidiales (MD = 0.05; p = 5.90 × 10^–04^), Monoblepharidales *incertae sedis* (MD = − 0.02; p = 1.40 × 10^–03^), and Trigonopsidaceae (MD = − 0.01; p = 1.10 × 10^–05^) were significantly more abundant in the Cascades.

Physiochemical measurements between the Cascade and Rocky Mountains (Table 1) show several significant differences, including for oxidation–reduction potential (ORP) (S = − 33; p = 0.007), dissolved oxygen (DO) (S = 39; p = 0.0005), Conductivity (S = 28.5; p = 0.04), NO_3_^−^ (S = − 38; p = 0.001) and pH (S = 23; p = 0.04); where ORP, NO_3_^−^ have higher values in the Cascade Mountains and DO, Conductivity, and pH have higher values in the Rocky Mountains. Diversity, evenness, and richness measurements between the Cascade and Rocky Mountains for algae, bacteria, fungi, and the whole community showed little difference (Supplementary Table S1).

**Table 1:** Wilcoxon Rank-Sum tests (S) of environmental features between snow algae blooms in the Rockies and Cascades. Mean environmental features are also shown for the Cascades (${\overline{\text{X}}}$_C_) and Rockies (${\overline{\text{X}}}$_R_).

Rockies–Cascades				
Environmental feature	S	P-value	${\overline{\text{X}}}$_C_	${\overline{\text{X}}}$_R_
**DO (%)**	**39**	**0**	43	57
**Conductivity (µS/cm)**	**28.5**	**0.02**	3.00	11.67
**TDS (ppm)**	**27.5**	**0.02**	1.50	5.92
Salinity (PSU)	11.5	0.25	0	0
NO_3_^−^ (mv)	− 38	1	118	83
NH_4_^+^ (mv)	− 9	0.74	− 214	− 220
K^+^ (mv)	17.5	0.09	− 204	− 197
Algae (cells/mL)	− 18	0.92	79,167	34,667
Pollen (grains/mL)	− 3.5	0.62	678	833
**pH**	**23**	**0.04**	6.74	7.64
ORP (mv)	− 33	1	16.22	− 6.40

Significant values are in [**bold**]

### Differences within snow algae blooms

**Figure 2:**
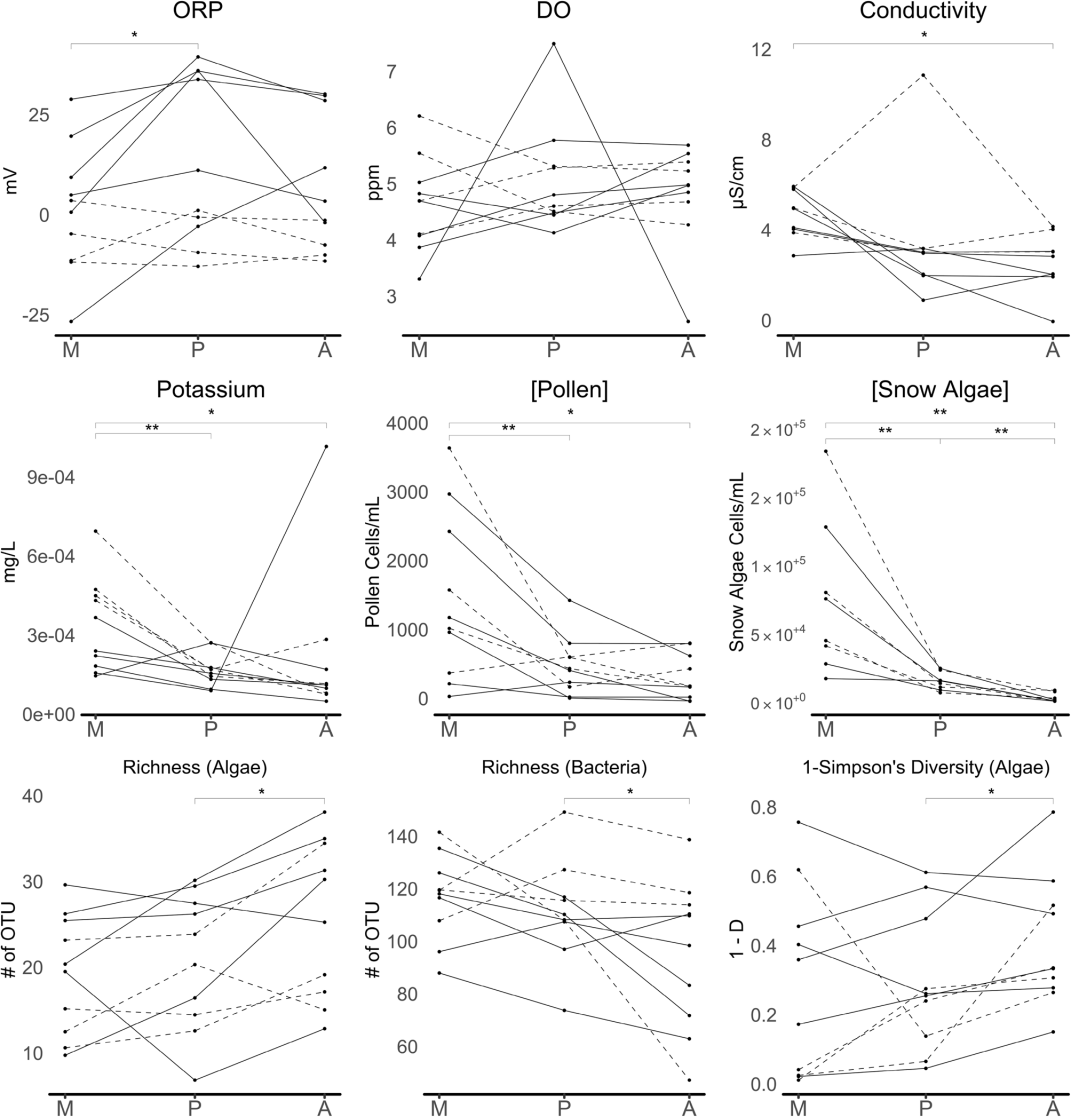
Parallel plots showing significantly different Wilcoxon Rank-Sum tests of environmental traits and diversity estimators between paired snow algae bloom zones M, P and A (* for p < 0.05 and ** for p < 0.01) and the Cascades and Rockies (solid and dashed lines respectively). Parameters not included were not found to have pairwise significance differences or may have been influenced by outliers. Even though not significant, DO was shown as M nearly met the cutoff for significance for between M-P and M-A (see text). Points represent individual samples.

By evaluating differences across zones within the same algae bloom, several interesting patterns emerge (Fig. 2). Unsurprisingly, the concentration of algal cells differed across zones (Wilcoxon rank-sum tests) with M-P (S = 22.5; p = 0.0039), M-A (S = 22.5; p = 0.0039) and P-A (S = 22.5; p = 0.0039), increasing from an average concentration of 2.10 × 10^3^ cells mL^−1^ in A, 1.40 × 10^4^ cells mL^−1^ in P, to 1.68 × 10^5^ cells mL^−1^ in M. Pollen concentrations also showed a similar pattern, differing between zones M-A (S = 23; p = 0.0078) and M-P (S = 23.5; p = 0.0078). Zones A and P had average concentrations of 320 and 420 grains mL^−1^ respectively, while zone M had an average concentration of 1.44 × 10^3^ grains mL^−1^.

We found significant pairwise zonal differences for electrochemical measures (Fig. 2). ORP was lower in M than in P (S = 21.5; p = 0.0137), while conductivity was significantly higher in M than A (S = 23; p = 0.0156). Dissolved oxygen (DO) levels were measured and, while not statistically significant (S = − 11.25; p = 0.1377), average DO levels did have lower and potentially biologically important average levels in M (46.85%) compared with P (50.8%) and A (49.35%). A significant increase in potassium levels was also observed in zone M compared to P (S = 23.5; p = 0.0137) and A (S = 17.5; p = 0.042).

Diversity estimates were also shown to differ across zones (Fig. 2). Between zones A and P, a decrease in algal richness (S = − 22.5; p = 0.0098) and Simpson’s Diversity (1-D; S = − 21.5; p = 0.0137) coincided with an increase in bacterial richness (S = 19.5; p = 0.0244). Algal richness was also shown to decrease from zone A to M (S = − 19.5; p = 0.0244).

As broad taxonomic differences were observed across snow algae bloom zones for algal and bacterial richness and for Simpson’s Diversity, we sought to identify specific community members responsible for these differences. To do so, we used Wilcoxon Rank-Sum tests across all zone pairs (M-P, M-A and P-A) at each taxonomic level, a subset of significant taxa is shown in Table 2. Relatively few OTUs differed between zones. *Sanguina nivaloides* had more sequence representation in P than A (MD = 0.096; p = 0.05), but not between M and P (MD = − 0.013; p = 0.58) or M and A (MD = 0.083; p = 0.14). Interestingly, a bacterial OTU within the genus *Polaromonas* was more abundant in M than A (MD = 0.02; p = 0.003) and in P than A (MD = 0.016; p = 0.011) suggesting an increased OTU relative abundance for *Sanguina* and *Polaromonas* in peripheral snow as compared to ‘white’ adjacent snows. For fungi, an unclassified Microbotryomycete had lower abundances in P than A (MD = − 0.03; p = 0.11). Pairwise Metacoder heat trees^43^ were generated to show how individual OTUs at varying taxonomic levels differ in terms of relative abundance between zones M, P, and A for the Cascade and Rocky Mountains (Supplementary Fig. S1).

**Table 2:** Significant OTU comparisons by snow zones medial (M), peripheral (P) and adjacent (A) at varying taxonomic levels (Wilcoxon Rank-Sum; p < 0.10).

Taxon	Zone 1	Zone 2	Median difference	Wilcoxon p-value
**Algae**				
*Sanguina nivaloides*	P	A	0.11	0.05
Viridiplantae	P	A	0.05	0.06
Chlorophyta	P	A	0.05	0.06
*Sanguina*	P	A	0.1	0.06
Chlorophyceae	P	A	0.07	0.11
Chlamydomonadales	P	A	0.07	0.11
Chlamydomonadaceae	P	A	0.07	0.11
**Bacteria**				
*Polaromonas*	M	A	0.01	0
*Polaromonas*	P	A	0.02	0.01
Gammaproteobacteria	M	A	0.02	0.02
Proteobacteria	M	A	0.02	0.04
Betaproteobacteriales	M	A	0.02	0.04
**Fungi**				
Burkholderiaceae	M	A	0.02	0.05
Microbotryomycetes	P	A	− 0.07	0.06
Fungi	P	A	− 0.06	0.08
Basidiomycota	P	A	− 0.05	0.08
Agaricomycetes unclassified	M	A	0.01	0.09
Microbotryomycetes unclassified	P	A	− 0.03	0.11

#### PerMANOVA

PerMANOVA tests for algae, bacteria, fungi, and the entire community (Table 3) show differential responses to environmental or physiochemical features. Algae showed no significant community differences with environmental traits, whereas bacterial communities did differ with oxidation–reduction potential (ORP) (R^2^ = 0.105; p = 0.05) and dissolved oxygen (DO) (R^2^ = 0.071; p = 0.044) and marginally with potassium (R^2^ = 0.062; p = 0.060). Fungi differed with ORP (R^2^ = 0.122; p = 0.005), pH (R^2^ = 0.169; p = 0.001), conductivity (R^2^ = 0.059; p = 0.003), TDS (R^2^ = 0.056; p = 0.013), nitrate (R^2^ = 0.078; p = 0.016), salinity (R^2^ = 0.05; p = 0.032), and potassium (R^2^ = 0.073; p = 0.043) and marginally with DO (R^2^ = 0.06; p = 0.073). Regional differences were observed for bacteria (R^2^ = 0.114; p = 0.011) and fungi (R^2^ = 0.154; p = 0.002). PerMANOVA tests using the whole community (all OTUs combined) showed significant between-group variation for ORP, region (R^2^ = 0.116; p = 0.007), pH (R^2^ = 0.109; p = 0.008) and potassium (R^2^ = 0.088; p = 0.025); nitrate (R^2^ = 0.058; p = 0.055) also likely plays a role but may only influence a sub-community of microbes (see below).

**Table 3:** PERMANOVA values for environmental traits of algal, bacterial, fungal, and community taxonomic groups. Bolded features were found to be significant (p < 0.05).

Algae					Bacteria				
	DF	Pseudo-F	R^2^	Prob > F		DF	Pseudo-F	R^2^	Prob > F
Potassium	1	3.13	0.1	0.06	**ORP**	**1**	**3.56**	**0.11**	**0.01**
pH	1	1.18	0.04	0.27	**Region**	**1**	**3.88**	**0.11**	**0.01**
TDS	1	0.84	0.03	0.36	**DO**	**1**	**2.41**	**0.07**	**0.04**
Conductivity	1	0.78	0.03	0.37	Potassium	1	2.08	0.06	0.06
ORP	1	0.87	0.03	0.4	pH	1	2.08	0.06	0.07
Pollen	1	0.86	0.03	0.42	Nitrate	1	1.73	0.05	0.12
Nitrate	1	0.8	0.03	0.46	Snow Algae	1	1.23	0.04	0.26
Region	1	0.74	0.03	0.48	Salinity	1	1.05	0.03	0.34
Salinity	1	0.59	0.02	0.53	Conductivity	1	1.01	0.03	0.37
Snow Algae	1	0.52	0.02	0.56	TDS	1	1.05	0.03	0.38
Ammonium	1	0.61	0.02	0.56	Pollen	1	0.99	0.03	0.4
DO	1	0.16	0.01	0.94	Ammonium	1	0.48	0.01	0.84

Fungi					Community				
	DF	Pseudo-F	R^2^	Prob > F		DF	Pseudo-F	R^2^	Prob > F
**pH**	**1**	**6.67**	**0.17**	**0**	**ORP**	**1**	**4.24**	**0.12**	**0.01**
**Region**	**1**	**6.08**	**0.15**	**0**	**Region**	**1**	**4.08**	**0.12**	**0.01**
**Conductivity**	**1**	**2.32**	**0.06**	**0**	**pH**	**1**	**3.84**	**0.11**	**0.01**
**ORP**	**1**	**4.84**	**0.12**	**0.01**	**Potassium**	**1**	**3.11**	**0.09**	**0.03**
**TDS**	**1**	**2.22**	**0.06**	**0.01**	Nitrate	1	2.05	0.06	0.06
**Nitrate**	**1**	**3.09**	**0.08**	**0.02**	DO	1	1.75	0.05	0.09
**Salinity**	**1**	**1.98**	**0.05**	**0.03**	Conductivity	1	1.39	0.04	0.19
**Potassium**	**1**	**2.88**	**0.07**	**0.04**	TDS	1	1.37	0.04	0.2
Pollen	1	2.52	0.06	0.06	Pollen	1	1.41	0.04	0.21
DO	1	2.36	0.06	0.07	Salinity	1	1.23	0.04	0.29
Snow Algae	1	1.06	0.03	0.44	Snow Algae	1	0.83	0.02	0.58
Ammonium	1	0.71	0.02	0.55	Ammonium	1	0.63	0.02	0.72

Significant values are in [**bold**]

### Taxonomic clusters versus environmental features (WOCNA analysis)

Weighted-OTU correlation network analyses (WOCNA) were used to assess if OTU sub-networks of red snow algae blooms respond to environmental features, nutrient concentrations, and to highly abundant taxa. By understanding taxonomic shifts and which microorganisms predominate we can begin to assess whether red snow algae blooms possess core taxa and whether OTU co-associations might suggest patterns of niche or trophic partitioning.

To assess whether sub-networks of OTUs (similarly responsive sub-communities) are structured across sampling sites, hierarchical clustering of OTUs was used to find stable modules. Seven modules were identified for the Cascade Mountains and eight for the Rocky Mountains (Supplementary Fig. S2). Each module set for the Cascade and Rocky Mountains had a single dominant module with high bootstrap stability, 37% and 32% respectively; remaining modules had fewer OTUs with lower stability.

**Figure 3:**
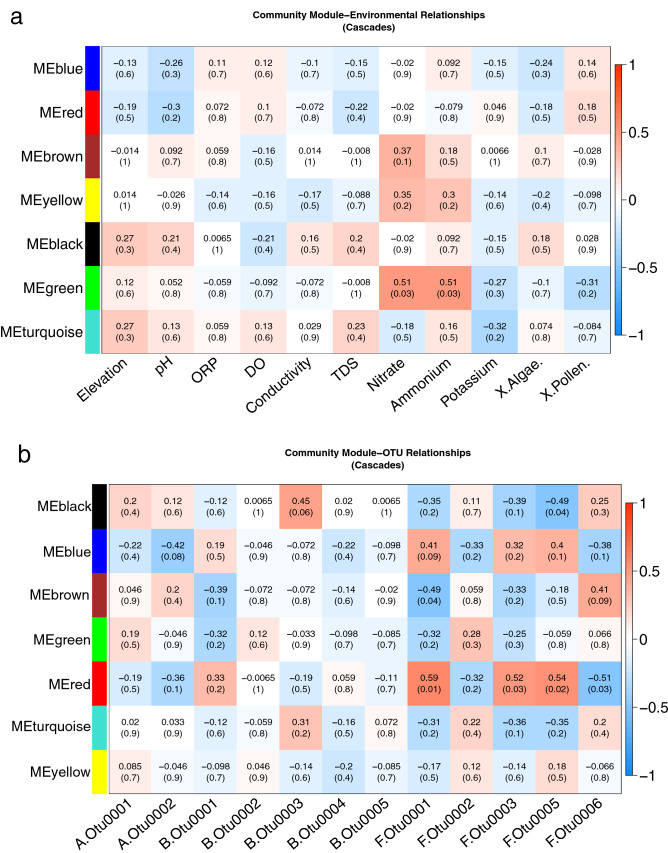
**(a, b)** Heat map showing snow community modules in the Cascades with (**a**) module-environment and (**b**) module-OTU correlations (Kendall-Tau). OTUs of interest were user selected and highly abundant. The upper value in each individual square is the correlation coefficient (with p-values parenthetical). Modules of the same color in different regions are not necessarily comprised of the same OTUs.

We tested if modules were correlated (Kendall-Tau) with environmental features and present these as heat maps (Fig. 3). In the Cascade Mountains, the strongest association are for the green module (Fig. 3a), which is negatively correlated with ammonium (K = 0.51; p = 0.03) and nitrate (K = 0.51; p = 0.03). The green module from the Cascades is comprised of several highly abundant OTUs best identified to the following genera: *Caldalkalibacillus* (BOTU0014 and BOTU0028), *Nesterenkonia* (BOTU0015 and BOTU0032), *Nakamurella* (BOTU0022), *Halomonas* (BOTU0026), Bacillaceae unclassified (BOTU0038) and the alga *Trebouxia* (AOTU0028). Interestingly, a near identical set of OTUs were also observed to cluster together in the Rocky Mountains black module (all except *Trebouxia*—AOTU0028) indicating similar OTU sub-network clusters form independently across regions. However, the Rocky Mountain black module did not show a relationship with ammonium or nitrite, as the near identical taxonomic module in the Cascade Mountains did.

Separate modules in the Rocky Mountains (Fig. 3b) showed weak to moderate associations with ammonium (red, green blue, yellow) and nitrate (yellow, green, blue), and stronger associations with DO, ORP and elevation. The red module was negatively correlated with DO (K = − 0.39; p = 0.2) and ORP (K = − 0.5; p = 0.09). Yellow and blue modules showed positive correlation with DO (K = 0.36; p = 0.2) (K = 0.27; p = 0.4) and ORP (K = 0.41; p = 0.2) (K = 0.63; p = 0.03) respectively; the yellow module was composed entirely of fungi and contained an abundant Microbotryomycete (FOTU0006) and unclassified fungi (FOTU0008); and the blue module was composed of abundant *Solitelia* (BOTU0001), *Rhizocarpon* (FOTU0023), and *Aureobasidium* (FOTU0032). Green and pink modules also showed negative correlation with elevation (K = − 0.7; p = 0.01) (K = − 0.5; p = 0.1).

**Figure 4:**
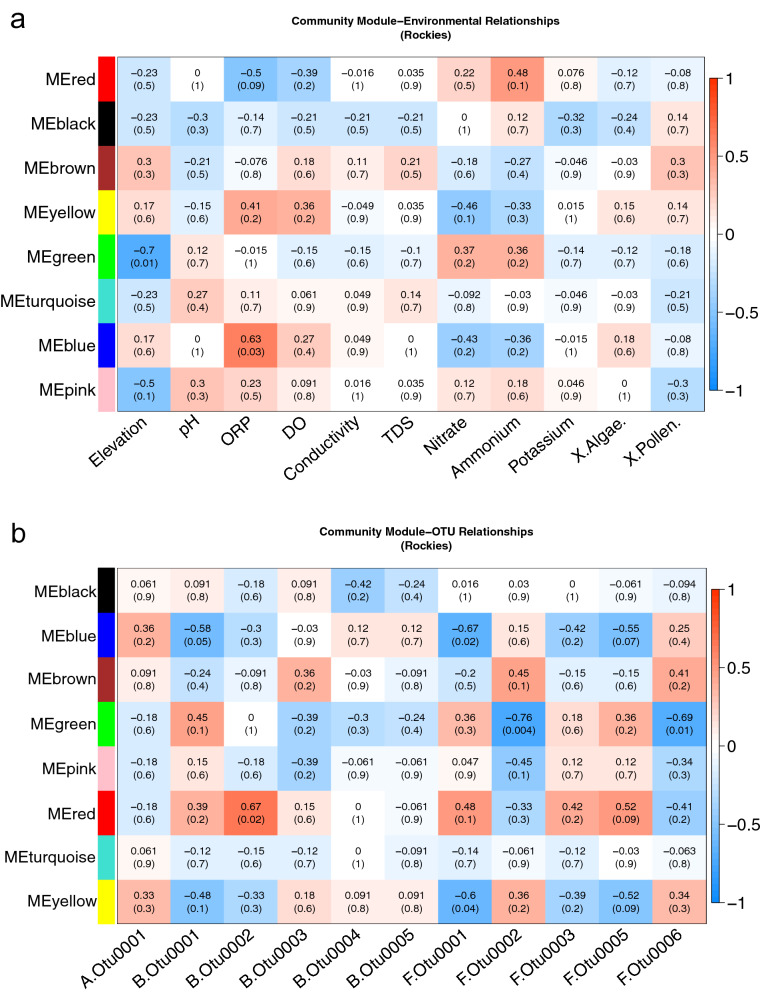
**(a, b)** Heat map showing snow community modules in the Rockies with (**a**) module-environment and (**b**) module-OTU correlations (Kendall-Tau). OTUs of interest were user selected and highly abundant. The upper value in each individual square is the correlation coefficient (with p-values parenthetical). Modules of the same color in different regions are not necessarily comprised of the same OTUs.

In addition to module-environment associations, highly abundant OTUs were parameterized to explore module-OTU associations for the Cascade and Rocky Mountains (Fig. 4). There are more significant associations between modules with fungal OTUs, compared with bacterial or algal OTUs, which suggests fungi in snow may be important for trophic function. For all modules in both regions, *Sanguina* sp*.* showed weak non-significant correlations. In the Cascades, the red module (N = 14) consisted entirely of fungi and included the highly abundant but unclassified chytrid (FOTU0001) and Leucosporidiales (FOTU0005) as well as less abundant Leucosporidiales and Microbotryomycetes. This red module showed moderate positive associations with the unclassified chytrid (FOTU0001) and two Leucosporidiales (FOTU0003 and FOTU0005) while showing moderate negative association with a Microbotryomycete (FOTU0006). It should be noted, modules in Figs. 3 and 4 are not necessarily composed of the same sets of OTUs.

### OTU co-association networks

Given the granular resolution of the WOCNA, first-order neighborhood OTU co-association networks were generated to assess relationships between abundant taxa and the entire OTU community. *S*. *nivaloides* had limited connectivity in the Cascades and Rocky Mountains; in strong contrast with the high connectivity and significant correlations between *Solitalea* sp. (BOTU0001) and unclassified Chytridiomycota (FOTU0001) (Supplementary Fig. S3). Of these positively correlated OTUs, several members of the order Leucosporidiales (FOTU0003, FOTU0005 and FOTU0008) were shown to positively and significantly co-associate with this chytrid. These OTUs were abundant and together comprise about 18% of the Cascades total OTU abundance. *Polaromonas* sp. (BOTU0004) had few associations in the Cascade Mountains but showed a robust and interconnected network in the Rocky Mountains (Supplementary Fig. S3), which associated with several highly abundant bacterial and fungal OTUs.

## Discussion

This study considers the connections of microbial communities and environmental features with density of algae within red snow algae blooms. To do this, algal cell density measures were categorized into visually discernable zones, M, P and A, representing a discrete density gradient. We show there is little evidence of biogeographic structuring across semi-continental distances for algae and bacteria, whereas fungi show regional structuring. Additionally, OTU communities in each zone show greater tendency to cluster with adjacent zones of local sample sites, rather than across sample sites with zones of the same category (Supplementary Fig. S4). This suggests that local sampling sites and environmental filtering may be more important than the algae bloom zones (M, P, and A) in determining community structure.

Regarding *S. nivaloides* (AOTU0001), network connections between it and other dominant bacteria and fungi (top 90%) are disjoint (Supplementary Fig. S3). The most abundant fungal OTU (FOTU0001) was an unclassified member of phylum Chytridiomycota and has high network connectivity with other abundant fungal and bacterial OTUs (Supplementary Fig. S3), but not with *S. nivaloides* (AOTU0001). This complicates suggestions that snow algae exclusively facilitate community formation and/or functionality^15^ and may support a hypothesis of disjunct and independent microbial sub-communities that co-occur in space and time, yet have limited interactions with algae in late-season snows. These sparsely connected networks support exploring whether carbon-based functional niche partitioning occurs between algae and pollen in snows. While this study did observe *Chlamydomonas reinhardtii* amplicons in snows, it is highly improbable these are *C. reinhardtii*; rather, it is likely that this best match against the global genetic databases alludes to the paucity of sequence database information for snow algae. This work also gives confirmation that a statistically significant signal can differentiate OTU relative abundances for *S. nivaloides* between zones P and A (Table 2).

We observed abundant bacterial taxa belonging to the classes ⍺-Proteobacteria, Actinobacteria, Bacilli, Bacteroidia and ɣ-Proteobacteria. Taxa within *Polaromonas,* which is abundant here, has been reported to be abundant within snow algae blooms^11^ and their trait diversification may be driven by adaptation to local conditions and algal hosts^44^. In the present study, *Polaromonas* OTU abundance increased concomitantly with algal density, suggesting snow algae blooms may facilitate niche expansion of select taxa, or conversely, snow algae acclimation may be facilitated by *Polaromonas sp*. Two WOCNA modules (green—Cascades, and black—Rockies) form hubs of OTUs that are nearly identical across regions at the genus level. These genera include *Caldalkalibacillus* (BOTU0014 and BOTU0028), *Nesterenkonia* (BOTU0015 and BOTU0032), *Nakamurella* (BOTU0022), *Halomonas* (BOTU0026), Bacillaceae unclassified (BOTU0038) and *Trebouxia* (AOTU0028; Rockies only), many of which have extremophilic relatives adapted to alkali-, halo-, psychro-, and/or thermophilic conditions^45–48^. None of these genera co-occurred with *S. nivaloides* (AOTU0001) nor were they found to vary significantly across zones. Given their abundance in the Cascade and Rocky Mountains, and the independent formation of near identical modules based on highly similar co-occurrence patterns in both localities, these taxa may occupy a discrete but currently unresolved niche.

We observed abundant fungal classes including Microbotryomycetes, Chytridiomycetes, Dothideomycetes and Monoblepharidomycetes. While observed biodiversity of fungi within snow algae blooms is high, the number of true cryobionts that grow and/or reproduce in this niche may be a smaller fraction of the OTUs observed, additional work to confirm physiological activity is needed.

Previous work indicates that Chytridiomycota can co-occur with snow algae^15,16,28^ and may parasitize *S. nivaloides* hypnozygotes, but not cysts^49^. In this study, Chytridiomycotan OTUs constituted the largest relative abundance of all OTU sequences (28%), which was higher in the Cascades (91%) than the Rockies (9%). Diversity of Chytridiomycota across both regions included OTUs best identified as: Chytridiomycota unclassified (~ 97%), *Hyaloraphidium curvatum* (3%), and *Rhizophydium* sp*.* (< 0.001%). The reason for the high abundance of these unresolved chytrids in the Cascade Mountains and the sparse representation in the Rocky Mountains is unclear, but may be due to different nutritional resources, possibly due to the lower elevation of the Cascade Mountain sampling locations; the presence of polyploidy or aneuploidy resulting in higher ITS2 copy numbers, a known feature of many Chytridiomycota^50^ and certain algae^51^. Earlier work^15,16^ has suggested that some snow chytrids are abundant in the absence of snow algae colonization, complicating the interpretation between snow chytrids and snow algae. Some chytrids are also known to be saprotrophs of Pinaceae pollen^52,53^, which was found in increasing concentrations from zones A to P to M. However, increases in pollen concentration were not associated with increases in chytrid OTU sequences abundances in this study, but the potential for pollen induction of chytrid populations should not be precluded based on this evidence. Chytridiomycota also showed a strong and significant positive co-association with Leucosporidiales, first demonstrated here, and both are known to frequently co-occur in snow, ice, and marine systems^15,54,55^. Future studies will help determine whether predominantly heterotrophic co-association networks are inducible by a common carbon source such as pollen or algae.

Certain taxa present in snow may also be in a continuum with the underlying soil^56^ and atmospheric^57^ microbial communities. These events are interspersed with continuous dry deposition that also infuse snows with nutrients. Interestingly, some pollen, fungal spores and bacteria may have evolved to have low supersaturation levels and/or wind transport potential^58^, which may increase the potential for bioorganic deposition. Genera found in an extensive atmospheric survey whose sampling locations partially overlapped with our snows include: *Alternaria*, *Aureobasidium*, *Didymella*, *Knufia*, *Mycosphaerella*, *Rhodotorula* and *Sistotrema*^59^; supporting the idea that atmospheric seeding of taxa in snow may occur but it is unclear if these taxa can become active in these snows.

Our study demonstrates that snow algae are associated with NO_3_^−^ and NH_4_^+^ across zones of algae density and that K^+^ appears to increase from zones A-M and P-M, having the highest concentrations in M. Given that K^+^ concentrations were highest in M and that two previous studies exploring fertilizer effects showed that NP fertilizer did not induce growth^11^ while NPK fertilizer did^35^, further exploration of the role of K^+^ in snow algae bloom development is warranted as it is likely limiting here, as has been demonstrated in other aquatic systems^60^. Moreover, low potassium levels have been linked to *Chytridium* sp*.* infections of some algae^61^.

Snow algae blooms typically occur in low conductivity environments^17^ where cell lysis through osmotic stress and increased freeze/thaw cycles during summer is thought to increase conductivity^62,63^. This study revealed significant differences in electrochemical measures in M compared to A, for ORP, conductivity and to some extents DO, suggesting higher microbial activity in zone M compared to A. However, it is unclear with these data if this difference is due to differential deposition of allochthonous material or differential release of organic and inorganic material. Also, as samples were collected only during the peak of bloom development, and bloom development started weeks prior to collection, further studies are necessary to understand physiochemical changes over time and if these observed results are similar across the growing season.

Bisaccate pollen grains were abundant and positively correlated with *S. nivaloides* cell counts. It is unclear, however, if pollen can facilitate snow algal bloom development via co-occurring saprotrophic nutrient release, can facilitate local competitive outcomes in snow algae communities via differential heterotrophic decomposition dynamics, or if this is merely incidental. Like Yakimovich, Engstrom and Quarmby 2020, we found *S. nivaloides* (AOTU0001) taxonomic networks lacking associations with bacteria, including dominant taxa such as *Solitalea* sp. (BOTU0001) and *Hymenobacter* sp. (BOTU0002)^27^. The same lack of associations with fungi was also apparent (Supplementary Fig. S3), which included Chytridiomycota unclassified (FOTU0001), unclassified Microbotryomycetes (FOTU0002), and Leucosporidiaceae (FOTU0003). Future spatiotemporal studies of snow algae bloom pulse/press dynamics are needed to discern trophic partitioning of algal and/or pollen primary production. Developing trophic indices specific to snow would enhance further investigations into this system.

We hypothesized that algae may be facilitating microbial community dynamics, but our pollen and co-association data leave open the possibility that pollen may be a driver of community diversity in snows, rather than algae. Separately, there is the question of whether pollen can facilitate algal bloom development or succession; if so, it is reasonable to expect they would overlap spatially. However, there are two main reasons why algae and pollen could show similar patterns but not be coupled: (1) airborne particulates, including non-pollen nutrient sources necessary to promote snow algae growth, are heterogeneously deposited to alpine snow during snow deposition and drifting events due to local topological variations leading to co-accumulation; and (2), snow melt dynamics causes an uneven distribution of biomaterial (including algae and pollen) that lead to snow algae blooms of varying intensity and minimal taxonomic differences across snow zones as shown here. Both scenarios may be possible since snow deposition, distribution, drifting and melt increase in variability over landscapes as spatial complexity increases^64^. Regional atmospheric concentrations of Pinaceae pollen (2003–2017) peaks in late-May in the Cascades (> 100 grains m^−3^) and in early-June in the Rockies (10–100 grains m^−3^) and extends through October^65^. While limited data exist documenting snow algae bloom onset, pollen peaks appear to coincide temporally with snow algae bloom development. To help clarify any relationship between pollen and snow algae, the authors recommend that future studies consider a quantitative pollen measure.

This study gives the first in depth look at the spatial differences of snow algae bloom communities and reveals environmental features and taxa that associate with differences in *S. nivaloides* concentrations. Our research suggests that snow algae blooms may have an active peripheral zone and a less active medial and adjacent zone that shift between autotrophic and heterotrophic maxima. This research also leads us to hypothesize that these zones may consist of reliable sub-OTU networks, which overlap in niche space and ultimately interact as discrete trophic networks anchored by either algae or pollen. Lastly, the concomitant increase of pollen with snow algae concentrations while observed and speculated to be of importance in previous studies^28,40,42,66,67^, but not typically examined or quantified, may be a crucial feature of future ecological studies of communities within snow algae blooms.

## Methods

### Sample collection

Red snow algae blooms were sampled in 2018 from all accessible blooms found at our sampling locations Glacier Peak Wilderness Area, Washington, USA (six independent blooms), and from the Arapaho and Medicine Bow-Routt National Forest, Wyoming and Colorado, USA (4 independent blooms). Each snow algae bloom was visually separated into three zones corresponding to algal density (Supplementary Fig. S5): medial (M), high density and deep red; peripheral (P), moderate density and pink; and adjacent (A), low to no density and white. Adjacent snow was visually uncolonized and 3 m from visible algal colonization. Samples (N = 30) were taken from sites in full sun, open landscapes, and above tree line. For each sample, GPS coordinates, elevation, and slope face were recorded (Supplementary Table S2) and all samples were visually free of apparent anthropogenic or animal disturbances. Ten volumetric (~ 85 cm^3^) subsamples were taken to a depth of ~ 5 cm from M, P and A for each bloom and placed into new 1-gallon zip-top plastic bags^15,16^. Collection devices were surface sanitized in the field using 70% denatured alcohol between samplings. Snows were melted at ambient temperature and homogenized. Snowmelt was dual filtered for enrichment of larger (algae and fungi; 2.0-μm) and smaller particles (bacteria; 0.22-μm)^16^. Nuclepore Membranes in Swin-Lok holders were used with a syringe and 100 mL of snowmelt was first passed through the 2.0-μm filter. Filtrate was collected into 500 mL polyethylene plastic bottles field-sanitized with denatured ethanol, then passed through the 0.22-μm filter. Membrane discs were transferred into Qiagen PowerBead Tubes with the addition of Buffer CD1 to prevent nucleotide degradation and stored on ice/snow until shipment, then stored at − 20 °C until gDNA extraction. In total, 60 samples were collected.

### Environmental variables

Melted snow samples were measured in the field for: ammonium (mV), conductivity (µS/cm), dissolved oxygen (ppm), oxidation–reduction potential (mV), pH, salinity (PSU), and total dissolved solids (ppm) using HI9829 Multiparameter Meter (Hanna Instruments, Woonsocket, RI, USA). Nitrate (NO_3_^−^) and potassium (K^+^) were measured with LAQUAtwin pocket meters (NO3-11 and K-11; Horiba Technologies, Kyoto, Japan). Due to low free nitrate and potassium concentrations, which are common to snowmelt^10,13^, mV values were recorded and converted into mg/L using ion selective electrodes (ISE) conversions from the software Logger Pro® 3. While measurements of free phosphorus were attempted, concentrations were below the instrument detection limit. Density of snow algae (cells mL^−1^) and pollen (grains mL^−1^) were determined hemocytometrically upon returning to the lab. Pollen grain counts only included distinctly bisaccate anemophilous pollen.

### Extraction and sequencing

DNA was extracted using the DNeasy PowerSoil® Kit using standard procedures with modifications. First, membrane filters were sonicated for 10 min in Buffer CD1 (DNeasy proprietary buffer) to maximize cell resuspension from filters then filters were removed. To maximize physical lysis as *Sanguina* cysts are difficult to break, 0.25 mL of sterile 1.0 mm zirconium oxide beads were added to extraction tubes and samples were beaten twice for 30 s at maximum speed using a Bead Mill 24 Homogenizer.

To generate amplicon sequencing libraries, a two-step PCR approach was used. Primary amplicon libraries for algae and fungi were generated by targeting the Internal Transcribed Spacer 2 (ITS2) of the rDNA region with the primers fITS7^68^ and ITS4^69^; these primers amplify snow algae and fungi concurrently^16,18^. For bacteria, we targeted the V4 hypervariable region of 16S rDNA with the modified primers 515f. and 806r^70–72^. Primary PCR primer constructs also include Nextera sequencing primers (nexF and nexR) as well as a range of ambiguous nucleotides (N[3-6]) and were constructed as follows: nexF-N[3-6]-fITS7 and nexR-N[3-6]-ITS4 for algae and fungi; and nexF-N[3-6]-515f and nexR-N[3-6]-806r for bacteria. PCRs were conducted in duplicate 25 μL reactions containing: 2 μL DNA template, 12.5 μL Phusion Green™ Hot Start II High-Fidelity Master Mix, 5.5 μL nuclease-free H_2_O, and 2.5 μL each of the forward and reverse primers (at 1 μM). PCR parameters were set for: initial denaturation (98 °C; 30 s); 25 cycles of denaturation (98 °C; 10 s), primer annealing (51 °C for algae/fungi and 52.5 °C for bacteria; 30 s), extension (72 °C; 40 s); final extension step (72 °C; 10 min). The ramp rate was 1.0 °C s^−1^ for annealing steps. All amplifications were visually confirmed with gel electrophoresis.

Secondary PCR was conducted using respective forward and reverse primer pairs P5-i5-overlap and P7-i7-overlap, comprised of unique dual-barcoded MIDs (i5 and i7), Illumina Adaptor sequence (P5 and P7) and partial overlap with the nexF and nexR sequences that acts as the annealing site for primers. Forward and reverse secondary primers were mixed to generate unique dual-barcoded primers. Multiplexed amplicons were generated by PCR in a 25 μL reaction containing: 2.5 μL primary PCR product, 12.5 μL Phusion Green Hot Start II High-Fidelity PCR Master Mix, 7.5 μL molecular grade nuclease-free H_2_O, and 2.5 μL of dual-barcoded primer mix (0.5 μM each primer). PCR parameters were set for: denaturation (98 °C; 30 s); 10 cycles of denaturation (98 °C; 20 s), primer annealing (50 °C; 20 s), and elongation (72 °C; 50 s); a final extension step (72 °C; 10 min). In total, 35 cycles were run. All amplification was confirmed visually and negative controls consisting of sterile water were extracted, amplified, and sequenced and remained free of contamination.

PCR products were cleaned using AxyPrep™ Mag PCR Clean-up kits using a 1:1 ratio of PCR product to beads^73^. Cleaned PCR products were quantified using a Qubit 3.0 fluorometer with Qubit^Ⓡ^ dsDNA HS assay kits and pooled at equal concentrations into separate eukaryotic and bacterial libraries and cleaned once again as above. Libraries were sequenced on a single Illumina MiSeq (300PE) reaction with 70% fungi/algae: 30% bacteria loading ratio at the Integrated Genomics Facility (Manhattan, KS, USA). Sequence data were demultiplexed using the unique i5 and i7 sequence combinations to produce fastq files for each experimental unit (Supplementary Table S3).

### Bioinformatics

Sequence processing was primarily done using *mothur* (v.1.42.0)^74^. Paired sequences were contiged then screened to remove ambiguous bases or those with a homopolymer length greater than 6 for fungi and algae, and 12 for bacteria. Primer sequences were trimmed using *cutadapt* (v.1.17). Bacterial sequences were aligned against *SILVA* (v132.8) and screened to exclude non-V4 regions. ITS sequences cannot be reliably globally aligned. Sequences were pre-clustered using pseudo-single linkage clustering^75^ and chimeras were identified and removed using *VSEARCH*. Bacterial sequences were taxonomically identified against the RDP training set (v.10) and eukaryotic sequences were identified against the UNITE non-redundant database (v.6) using a Naïve Bayesian Classifier and off-target sequences were removed. As UNITE is a fungal database with limited non-fungal eukaryotes, non-fungal sequences were retained so algal subsets could be subsequently identified (BLASTn). For bacteria, an uncorrected pairwise distance matrix was determined (not punishing terminal gaps) between aligned sequences. The resultant distance matrix was used for OTU demarcation at a 3% dissimilarity threshold using OptiClust^76^. For algae and fungi, OTUs were demarcated using an abundance based *VSEARCH*. For all lineages, OTUs with fewer than 10 sequences were removed^77^. Consensus taxonomic identifications and representative sequences for each OTU were generated and separate OTU x sample matrices were created for algae, bacteria, and fungi. After sequence quality control, total sequence counts were: 2,094,357 for algae, 3,178,201 for bacteria and 2,632,246 for fungi. In total, 91 algal, 224 bacterial, 532 fungal OTUs were demarcated. A set of OTUs, comprising the entire community, was also compiled (847 OTUs). A BLAST hit tables of the top 10 hits for the top 10 most abundant OTUs for algae, bacteria, and fungi can be found in Supplementary Table S4.

### Statistical analysis

OTU richness (S_obs_), Simpson’s evenness (E_D_), Gini-Simpson’s diversity (1 − D), and Bray–Curtis dissimilarity values were calculated for algae, bacteria, fungi, and the whole community using an iterative subsampling approach and a subsampling depth was set to smallest sample size for algae (10,609), bacteria (50,542), fungi (29,950) and whole community (129,506) (1000 iterations) and mean values were used for all analyses. The prefix F, B, and A before OTU identities indicate if these are fungal, bacterial, or algal OTUs respectively.

To visualize community membership, relative abundance stacked histograms were generated in R (v3.4.5). Lineages corresponding to zones M, P and A from the Cascades and Rockies are shown, at the family (bacteria and fungi) or species (algae) level. Lineages were derived from OTU taxonomic assignments. Taxa with relative abundances of less than 1% were combined into a single group. Wilcoxon Rank-Sum tests were used 1) to test whether highly abundant OTUs differed between the Cascade and Rocky Mountains, and 2), to test whether environmental features differed between the Cascade and Rocky Mountains (Table 1).

A permutational multivariate analysis of variance (PerMANOVA)^78^ was run (R package *vegan*) on average Bray–Curtis values to test if communities of algae, bacteria, fungi, or the entire community differ with physiochemical (NH_4_^+^, DO, NO_3_^−^, ORP, pH, K^+^, and TDS), regional or biological (pollen and algae concentration) features.

Pairwise Wilcoxon Sign-Rank tests were used to test if environmental features and diversity estimates differ between snow zone pairs (M-P, M-A and P-A) and to generate parallel plots in R. Pairwise Wilcoxon Sign-Rank tests were conducted using JMP® Pro (v.14).

Weighted-OTU correlation network analyses (WOCNA) were conducted on the entire community OTU matrix from the Cascades and Rockies separately (as PerMANOVA tests indicated significant regional differences) using the *WGCNA* package^79,80^ with optimizations^81^. WOCNA was used to assess if community wide OTU modules correlate with environmental features. OTU modules are statistical interpretations presumed to respond to similar sets of environmental features. All WOCNA tests and parameters are in Supplementary Table S5.

Modifications to the WOCNA analyses were done for optimization for use with OTU data; raw counts were transformed to Hellinger distances, which give higher weight to more abundant OTUs; modules were assessed for stability using clusterwise Jaccard similarities, similar to SABRE^82^; correlations between modules and environmental features and abundant OTUs were assessed using Kendall-Tau correlations and heatmaps were generated (Figs. 3, 4). Module MDS plots are shown in Supplementary Figure S6.

Module vs. abundant OTU correlations were explored under the assumption that nutrient competition, resource sharing or saprotrophic utilization by groups of individual snow microbes underlie these associations. Correlations give an idea for how OTU consortia with similar relative abundance sample patterns associate with environmental features or other OTUs. Kendall-Tau co-association first-order neighborhood network graphs were generated in R for select OTUs from the Cascades and Rockies. Only vertices with K ≥|0.40| and p-value ≤ 0.1 were incorporated into the subnetworks. Networks were constrained by OTU absolute abundance (AA > 100) and by region.

## Supplementary Information

Supplementary Information.

## Data Availability

*Sequence Accessions—*sequence data is archived at the Sequence Read Archive (SRA) at NCBI under the accessions: BioProject (PRJNA730900) and BioSamples (SAMN19244874-SAMN19244994). *Code—*All code used to generate figures and analyze data has been stored in the following repository: https://github.com/tuck82er/snow.algae.bloom.2018.git.
