# Supplementary material for: Sampling a gradient of red snow algae bloom density reveals novel connections between microbial communities and environmental features
Source: Sci Rep. 2022 Jun 22;12:10536. doi: 10.1038/s41598-022-13914-7 (PMC9217940; doi:10.1038/s41598-022-13914-7)
Supplement: Supplementary file 1 — Supplementary Information. [file 41598_2022_13914_MOESM1_ESM.pdf]

Sampling a gradient of red snow algae bloom density reveals novel connections between microbial communities and environmental features

Avery E. Tucker[1,2*], Shawn P. Brown[1,2]

1: Department of Biological Sciences, The University of Memphis, Memphis, TN USA 38152

2: Center for Biodiversity Research, The University of Memphis, Memphis, TN USA 38152

Supplementary Table S1 Wilcoxon Rank-Sum tests of algae, bacteria, fungi for community diversity estimators between the Rockies and Cascades. Negative test statistics (S) indicate lower numeric values for estimators in the Rockies while positive values indicate the opposite. One-tailed p-values are shown.

	Algae		Bacteria		Fungi		Community	
	Test Statistic (S)	Prob > S	Test Statistic (S)	Prob > S	Test Statistic (S)	Prob > S	Test Statistic (S)	Prob > S
1-Simpson's Diversity	-17.00	0.90	17.00	0.10	16.00	0.12	-2.00	0.55
Evenness	2.00	0.45	7.00	0.31	7.00	0.31	2.00	0.45
Richness	-13.00	0.83	20.00	0.06	**25.00**	**0.03**	8.00	0.28

Supplementary Table S2 Environmental parameter data from all sampled locations according to region and zone.

Sample ID	Region	Zone	Slope Face	Altitude (mASL)	Latitude	Longitude	Algae Bray Mean	Bac Bray Mean	Fungi Bray Mean	Algae (Cells/mL)	Pollen (Pollen/mL)
R_M1	Rockies	M	E	3950	41º 20.796 N	106º 19.812 W	0.34	0.42	0.76	40000	400
R_P1	Rockies	P	E	3950	41º 20.796 N	106º 19.812 W	0.34	0.4	0.69	10000	600
R_A1	Rockies	A	E	3950	41º 20.796 N	106º 19.812 W	0.35	0.51	0.68	8000	800
R_M2	Rockies	M	W	3549	41º 21.578 N	106º 19.104 W	0.48	0.42	0.74	183000	1000
R_P2	Rockies	P	W	3549	41º 21.578 N	106º 19.104 W	0.32	0.39	0.68	23000	400
R_A2	Rockies	A	W	3549	41º 21.578 N	106º 19.104 W	0.34	0.41	0.67	7000	200
R_M3	Rockies	M	E	3633	41º 21.342 N	106º 17.700 W	0.33	0.36	0.66	44000	1600
R_P3	Rockies	P	E	3633	41º 21.342 N	106º 17.700 W	0.33	0.39	0.67	6000	200
R_A3	Rockies	A	E	3633	41º 21.342 N	106º 17.700 W	0.46	0.37	0.66	2000	400
R_M4	Rockies	M	E	3280	39º 20.384 N	106º 07.753 W	0.34	0.39	0.63	80000	3600
R_P4	Rockies	P	E	3280	39º 20.384 N	106º 07.753 W	0.33	0.37	0.65	13000	600
R_A4	Rockies	A	E	3280	39º 20.384 N	106º 07.753 W	0.35	0.36	0.63	0	200
C_M5	Cascades	M	E	1818	48º 10.32 N	120º 53.51 W	0.4	0.34	0.6	128000	2400
C_P5	Cascades	P	E	1818	48º 10.32 N	120º 53.51 W	0.68	0.42	0.57	24000	800
C_A5	Cascades	A	E	1818	48º 10.32 N	120º 53.51 W	0.68	0.37	0.57	1000	800
C_M6	Cascades	M	E	1825	48º 10.229 N	120º 53.058 W	0.79	0.36	0.59	75000	3000
C_P6	Cascades	P	E	1825	48º 10.229 N	120º 53.058 W	0.53	0.34	0.58	15000	1400
C_A6	Cascades	A	E	1825	48º 10.229 N	120º 53.058 W	0.65	0.36	0.59	0	600
C_M7	Cascades	M	N	2091	48º 10.234 N	120º 53.033 W	0.34	0.36	0.61	24000	1000
C_P7	Cascades	P	N	2091	48º 10.234 N	120º 53.033 W	0.33	0.39	0.62	11000	0
C_A7	Cascades	A	N	2091	48º 10.234 N	120º 53.033 W	0.33	0.37	0.62	0	0
C_M8	Cascades	M	N	2091	48º 10.139 N	120º 52.936 W	0.62	0.38	0.55	1.00E+06	1200
C_P8	Cascades	P	N	2091	48º 10.139 N	120º 52.936 W	0.62	0.41	0.65	15000	400
C_A8	Cascades	A	N	2091	48º 10.139 N	120º 52.936 W	0.6	0.39	0.55	3000	0
C_M9	Cascades	M	S	2127	48º 09.668 N	120º 52.714 W	0.33	0.4	0.6	28000	200
C_P9	Cascades	P	S	2127	48º 09.668 N	120º 52.714 W	0.33	0.35	0.54	8000	0
C_A9	Cascades	A	S	2127	48º 09.668 N	120º 52.714 W	0.35	0.36	0.56	0	0
C_M10	Cascades	M	S	1886	48º 10.42 N	120º 53.57 W	0.34	0.46	0.74	17000	0
C_P10	Cascades	P	S	1886	48º 10.42 N	120º 53.57 W	0.38	0.36	0.62	15000	200
C_A10	Cascades	A	S	1886	48º 10.42 N	120º 53.57 W	0.64	0.39	0.62	0	200

Supplementary Table S2 (Cont.) Environmental parameter data from all sampled locations according to region and zone. Variables considered outliers in analysis are bolded.

Sample ID	Region	Zone	Date	pH	ORP (mv)	DO (%)	DO (ppm)	Conductivity (μS/cm)	TDS (ppm)	Salinity (PSU)	NO_3^- (mg/L)	NH_4^+ (mg/L)	K^+ (mg/L)
R_M1	Rockies	M	12-Jul-18	6.6	3.5	66.1	6.21	5	3	0	9.16E+00	6.42E-05	4.72E-04
R_P1	Rockies	P	12-Jul-18	6.41	-0.6	63.9	5.32	3	2	0	1.39E+01	4.99E-04	1.43E-04
R_A1	Rockies	A	12-Jul-18	6.18	-1.3	63.8	5.23	4	2	0	1.23E+01	1.26E-04	1.16E-04
R_M2	Rockies	M	15-Jul-18	8.59	-11.5	51.9	4.7	6	3	0	7.75E+00	5.20E-04	6.92E-04
R_P2	Rockies	P	15-Jul-18	9.28	1.1	59.8	5.28	11	5	0	1.86E+01	3.43E-06	2.72E-04
R_A2	Rockies	A	15-Jul-18	9.13	-7.5	56.9	5.39	4	2	0	1.71E+01	5.66E-05	7.90E-05
R_M3	Rockies	M	15-Jul-18	8.35	-11.9	55.7	5.54	16	8	0.01	4.67E+01	1.91E-04	4.52E-04
R_P3	Rockies	P	15-Jul-18	8.29	-12.9	51.7	4.51	4	2	0	1.71E+01	1.11E-04	1.70E-04
R_A3	Rockies	A	15-Jul-18	7.61	-10.1	53	4.27	**77**	**39**	0.04	2.10E+03	1.99E-06	2.83E-04
R_M4	Rockies	M	15-Jul-18	7.17	-4.7	51.2	4.11	4	2	0	4.67E+01	1.11E-05	4.34E-04
R_P4	Rockies	P	15-Jul-18	7.08	-9.4	55.6	4.6	3	1	0	1.51E+01	4.79E-05	1.70E-04
R_A4	Rockies	A	15-Jul-18	6.93	-11.5	57.8	4.68	3	2	0	4.30E+01	5.66E-04	7.57E-05
C_M5	Cascades	M	12-Aug-18	5.37	28.8	47.4	5.02	4	2	0	8.08E+00	1.68E-03	1.43E-04
C_P5	Cascades	P	12-Aug-18	5.51	33.8	50.7	5.78	3	2	0	4.89E+00	3.02E-03	2.72E-04
C_A5	Cascades	A	12-Aug-18	5.56	29.8	49	5.69	3	1	0	4.89E+00	4.99E-06	1.70E-04
C_M6	Cascades	M	12-Aug-18	5.39	19.6	39.5	4.08	4	2	0	1.51E+01	8.97E-06	2.39E-04
C_P6	Cascades	P	12-Aug-18	5.7	35.9	45.4	4.8	3	2	0	4.32E+00	4.22E-05	1.77E-04
C_A6	Cascades	A	12-Aug-18	5.98	30.2	47.4	4.98	3	2	0	5.10E+00	1.20E-03	9.78E-05
C_M7	Cascades	M	12-Aug-18	6.74	5	43.4	4.7	3	1	0	5.10E+00	6.15E-06	2.19E-04
C_P7	Cascades	P	12-Aug-18	6.99	11.1	38.7	4.13	3	1	0	6.03E+00	1.75E-05	1.56E-04
C_A7	Cascades	A	12-Aug-18	6.61	3.4	47	4.97	2	1	0	3.97E+00	1.36E-04	1.11E-04
C_M8	Cascades	M	12-Aug-18	7.14	9.4	35.1	3.87	5	2	0	4.32E+00	5.43E-05	3.66E-04
C_P8	Cascades	P	12-Aug-18	8.08	39.4	37.4	4.48	2	1	0	5.10E+00	7.91E-04	1.32E-04
C_A8	Cascades	A	12-Aug-18	6.53	28.6	47.3	4.85	2	1	0	8.08E+00	4.05E-05	1.11E-04
C_M9	Cascades	M	12-Aug-18	7.46	-26.7	45.5	4.83	6	3	0	9.16E+00	1.61E-05	1.85E-04
C_P9	Cascades	P	12-Aug-18	8.03	-2.9	42.6	4.45	1	1	0	3.50E+00	4.05E-05	9.37E-05
C_A9	Cascades	A	12-Aug-18	8.47	11.8	43.1	5.54	2	1	0	5.79E+00	1.11E-05	4.95E-05
C_M10	Cascades	M	12-Aug-18	8.98	0.6	32.7	3.31	6	3	0	7.13E+00	8.25E-06	1.56E-04
C_P10	Cascades	P	12-Aug-18	5.37	36	62.2	7.5	2	1	0	9.96E+00	4.79E-04	8.98E-05
C_A10	Cascades	A	12-Aug-18	7.45	-1.9	28.2	2.55	0	0	0	1.45E+01	3.72E-03	1.02E-03

Supplementary Table S3 Primer sequences and MID (i5 and i7) sequences used for parsing sequences into experimental units.

Primary PCR Primers (1°)

**Fungi**	
nexF-N3-fITS7	TCGTCGGCAGCGTCAGATGTGTATAAGAGACAG-NNN-GTGARTCATCGAATCTTTG
nexF-N4-fITS7	TCGTCGGCAGCGTCAGATGTGTATAAGAGACAG-NNNN-GTGARTCATCGAATCTTTG
nexF-N5-fITS7	TCGTCGGCAGCGTCAGATGTGTATAAGAGACAG-NNNNN-GTGARTCATCGAATCTTTG
nexF-N6-fITS7	TCGTCGGCAGCGTCAGATGTGTATAAGAGACAG-NNNNNN-GTGARTCATCGAATCTTTG
nexF-N3-ITS4	GTCTCGTGGGCTCGGAGATGTGTATAAGAGACAG-NNN-TCCTCCGCTTATTGATATGC
nexF-N4-ITS4	GTCTCGTGGGCTCGGAGATGTGTATAAGAGACAG-NNNN-TCCTCCGCTTATTGATATGC
nexF-N5-ITS4	GTCTCGTGGGCTCGGAGATGTGTATAAGAGACAG-NNNNN-TCCTCCGCTTATTGATATGC
nexF-N6-ITS4	GTCTCGTGGGCTCGGAGATGTGTATAAGAGACAG-NNNNNN-TCCTCCGCTTATTGATATGC
**Bacteria**	
nexF-N3-515f	TCGTCGGCAGCGTCAGATGTGTATAAGAGACAG-NNN-GTGYCAGCMGCCGCGGTAA
nexF-N4-515f	TCGTCGGCAGCGTCAGATGTGTATAAGAGACAG-NNNN-GTGYCAGCMGCCGCGGTAA
nexF-N5-515f	TCGTCGGCAGCGTCAGATGTGTATAAGAGACAG-NNNNN-GTGYCAGCMGCCGCGGTAA
nexF-N6-515f	TCGTCGGCAGCGTCAGATGTGTATAAGAGACAG-NNNNNN-GTGYCAGCMGCCGCGGTAA
nexF-N3-806r	GTCTCGTGGGCTCGGAGATGTGTATAAGAGACAG-NNN-GGACTACNVGGGTWTCTAAT
nexF-N4-806r	GTCTCGTGGGCTCGGAGATGTGTATAAGAGACAG-NNNN-GGACTACNVGGGTWTCTAAT
nexF-N5-806r	GTCTCGTGGGCTCGGAGATGTGTATAAGAGACAG-NNNNN-GGACTACNVGGGTWTCTAAT
nexF-N6-806r	GTCTCGTGGGCTCGGAGATGTGTATAAGAGACAG-NNNNNN-GGACTACNVGGGTWTCTAAT

Secondary PCR Primers (2°)

P5-i5-Overlap	AATGATACGGCGACCACCGAGATCTACAC-i5-TCGTCGGCAGCGTC
P7-i7-Overlap	CAAGCAGAAGACGGCATACGAGAT-i7-GTCTCGTGGGCTCGG

Sample Name	Forward MID 5' - 3' (i5)	Reverse MID 5' - 3' (i7)
Fungi_R_M1_A	GGCCATAT	AGAGACAC
Fungi_R_P1_A	AGTGGTGA	AGAGACAC
Fungi_R_A1_A	GTGTTCTC	AGAGACAC

Fungi_R_M2_A	TGAGGACA	AGAGACAC
Fungi_R_P2_A	TTCGATGG	AGAGACAC
Fungi_R_A2_A	GTGTCACA	AGAGACAC
Fungi_R_M3_A	ACGTGATC	AGAGACAC
Fungi_R_P3_A	AGAGCAGT	AGAGACAC
Fungi_R_A3_A	CTCTAGAG	AGAGACAC
Fungi_R_M4_A	AACCGGTT	AGAGACAC
Fungi_R_P4_A	TGGTCAAC	AGAGACAC
Fungi_R_A4_A	GTACGATC	AGAGACAC
Fungi_C_M5_A	CACTTCTG	ACCTTGCT
Fungi_C_P5_A	GTAGAGGT	ACCTTGCT
Fungi_C_A5_A	CAGTCTCT	ACCTTGCT
Fungi_C_M6_A	ATCGGCAT	ACCTTGCT
Fungi_C_P6_A	ATGGCCTA	ACCTTGCT
Fungi_C_A6_A	AGTCTGTG	ACCTTGCT
Fungi_C_M7_A	AACCTTCC	ACCTTGCT
Fungi_C_P7_A	ACCTGTTC	ACCTTGCT
Fungi_C_A7_A	CAGACTCA	ACCTTGCT
Fungi_C_M8_A	AGTGTCTG	ACCTTGCT
Fungi_C_P8_A	CTTGGTAG	ACCTTGCT
Fungi_C_A8_A	TATAGCGC	ACCTTGCT
Fungi_C_M9_A	GTCACAGT	TTGCTACC
Fungi_C_P9_A	CTGACAGT	TTGCTACC
Fungi_C_A9_A	CTCTACAC	TTGCTACC
Fungi_C_M10_A	CAGTGACT	TTGCTACC
Fungi_C_P10_A	CTGTGTCT	TTGCTACC
Fungi_C_A10_A	TGGTACCA	TTGCTACC
Fungi_H2O_A	TCCTTGCA	TTGCTACC
Fungi_R_M1_B	TGGTGAAG	TTCCATGC
Fungi_R_P1_B	CGCGATTA	TTCCATGC
Fungi_R_A1_B	AGACGACA	TTCCATGC
Fungi_R_M2_B	TCTCAGAG	TTCCATGC
Fungi_R_P2_B	AGACGTCT	TTCCATGC
Fungi_R_A2_B	CGCGTATA	TTCCATGC
Fungi_R_M3_B	TGCTTGGA	TTCCATGC
Fungi_R_P3_B	TCCTTCCT	TTCCATGC
Fungi_R_A3_B	GGCCATAT	GTAGACCT
Fungi_R_M4_B	GACAACTC	TTCCATGC
Fungi_R_P4_B	CCAATTCC	TTCCATGC
Fungi_R_A4_B	CAAGCTAC	TTCCATGC
Fungi_C_M5_B	GTGACTGT	AACGAACG
Fungi_C_P5_B	CTGATCTC	AACGAACG
Fungi_C_A5_B	GATCCTAG	AACGAACG
Fungi_C_M6_B	TCCTAGGA	AACGAACG
Fungi_C_P6_B	CAACTGCA	AACGAACG
Fungi_C_A6_B	TACGGCTA	AACGAACG
Fungi_C_M7_B	CACTTGTC	AACGAACG

Fungi_C_P7_B	AGTCCACA	AACGAACG
Fungi_C_A7_B	TCTGGAGT	AACGAACG
Fungi_C_M8_B	CACTTCTG	AACGAACG
Fungi_C_P8_B	TTCCAAGG	AACGAACG
Fungi_C_A8_B	CTCACACT	AACGAACG
Fungi_C_M9_B	CGCCTTAT	GTACCAAC
Fungi_C_P9_B	AACGATCC	GTACCAAC
Fungi_C_A9_B	CTTGTGCT	GTACCAAC
Fungi_C_M10_B	TAGGCCTA	GTACCAAC
Fungi_C_P10_B	GTAGCTTG	GTACCAAC
Fungi_C_A10_B	AACCATCG	GTACCAAC
Fungi_H2O_B	TGGACTAC	GTACCAAC
Bacteria_R_M1_A	GGCCATAT	CGTTCCTA
Bacteria_R_P1_A	AGTGGTGA	CGTTCCTA
Bacteria_R_A1_A	GTGTTCTC	CGTTCCTA
Bacteria_R_M2_A	TGAGGACA	CGTTCCTA
Bacteria_R_P2_A	TTCGATGG	CGTTCCTA
Bacteria_R_A2_A	GTGTCACA	CGTTCCTA
Bacteria_R_M3_A	ACGTGATC	CGTTCCTA
Bacteria_R_P3_A	AGAGCAGT	CGTTCCTA
Bacteria_R_A3_A	CTCTAGAG	CGTTCCTA
Bacteria_R_M4_A	AACCGGTT	CGTTCCTA
Bacteria_R_P4_A	TGGTCAAC	CGTTCCTA
Bacteria_R_A4_A	GTACGATC	CGTTCCTA
Bacteria_C_M5_A	CACTTCTG	TGTGTGAC
Bacteria_C_P5_A	GTAGAGGT	TGTGTGAC
Bacteria_C_A5_A	CAGTCTCT	TGTGTGAC
Bacteria_C_M6_A	ATCGGCAT	TGTGTGAC
Bacteria_C_P6_A	ATGGCCTA	TGTGTGAC
Bacteria_C_A6_A	AGTCTGTG	TGTGTGAC
Bacteria_C_M7_A	AACCTTCC	TGTGTGAC
Bacteria_C_P7_A	ACCTGTTC	TGTGTGAC
Bacteria_C_A7_A	CAGACTCA	TGTGTGAC
Bacteria_C_M8_A	AGTGTCTG	TGTGTGAC
Bacteria_C_P8_A	CTTGGTAG	TGTGTGAC
Bacteria_C_A8_A	TATAGCGC	TGTGTGAC
Bacteria_C_M9_A	GTCACAGT	GAGTAGAC
Bacteria_C_P9_A	CTGACAGT	GAGTAGAC
Bacteria_C_A9_A	CTCTACAC	GAGTAGAC
Bacteria_C_M10_A	CAGTGACT	GAGTAGAC
Bacteria_C_P10_A	CTGTGTCT	GAGTAGAC
Bacteria_C_A10_A	TGGTACCA	GAGTAGAC
Bacteria_H2O_A	TCCTTGCA	GAGTAGAC
Bacteria_R_M1_B	TGGTGAAG	CAAGCAAG
Bacteria_R_P1_B	CGCGATTA	CAAGCAAG
Bacteria_R_A1_B	AGACGACA	CAAGCAAG
Bacteria_R_M2_B	TCTCAGAG	CAAGCAAG

Bacteria_R_P2_B	AGACGTCT	CAAGCAAG
Bacteria_R_A2_B	CGCGTATA	CAAGCAAG
Bacteria_R_M3_B	TGCTTGGA	CAAGCAAG
Bacteria_R_P3_B	TCCTTCCT	CAAGCAAG
Bacteria_R_A3_B	GGCCATAT	CAAGCAAG
Bacteria_R_M4_B	GACAACTC	CAAGCAAG
Bacteria_R_P4_B	CCAATTCC	CAAGCAAG
Bacteria_R_A4_B	CAAGCTAC	CAAGCAAG
Bacteria_C_M5_B	GTGACTGT	ATCGTTCC
Bacteria_C_P5_B	CTGATCTC	ATCGTTCC
Bacteria_C_A5_B	GATCCTAG	ATCGTTCC
Bacteria_C_M6_B	TCCTAGGA	ATCGTTCC
Bacteria_C_P6_B	CAACTGCA	ATCGTTCC
Bacteria_C_A6_B	TACGGCTA	ATCGTTCC
Bacteria_C_M7_B	CACTTGTC	ATCGTTCC
Bacteria_C_P7_B	AGTCCACA	ATCGTTCC
Bacteria_C_A7_B	TCTGGAGT	ATCGTTCC
Bacteria_C_M8_B	CACTTCTG	ATCGTTCC
Bacteria_C_P8_B	TTCCAAGG	ATCGTTCC
Bacteria_C_A8_B	CTCACACT	ATCGTTCC
Bacteria_C_M9_B	CGCCTTAT	ACTCTGTC
Bacteria_C_P9_B	AACGATCC	ACTCTGTC
Bacteria_C_A9_B	CTTGTGCT	ACTCTGTC
Bacteria_C_M10_B	TAGGCCTA	ACTCTGTC
Bacteria_C_P10_B	GTAGCTTG	ACTCTGTC
Bacteria_C_A10_B	AACCATCG	ACTCTGTC
Bacteria_H2O_B	TGGACTAC	ACTCTGTC

Supplementary Table S4 BLAST hit table for algae, bacteria, and fungi

OTU ID	ACCESSION	PER. IDENT.	BITS	GAPS	NUM. MATCH SEQ.	SEQ. START	SEQ. END	E-VALUE	SCORE
AOTU0001	LC381736.1	100	242	0	1	54	295	2.12E-121	448
AOTU0001	KX063743.1	100	241	0	2	1	241	7.64E-121	446
AOTU0001	AB902971.1	99.587	242	0	1	2077	2318	9.88E-120	442
AOTU0001	GU117577.1	99.587	242	0	1	2140	2381	9.88E-120	442
AOTU0001	KX063763.1	99.585	241	0	2	1	241	3.55E-119	440
AOTU0001	KX063744.1	99.585	241	0	2	1	241	3.55E-119	440
AOTU0001	AB902998.1	99.174	242	0	1	2111	2352	1.28E-118	438
AOTU0001	LC648245.1	99.174	242	0	1	2091	2332	4.60E-118	436
AOTU0001	KX063757.1	99.17	241	0	2	1	241	1.65E-117	435
AOTU0001	KX063746.1	99.17	241	0	2	1	241	1.65E-117	435
AOTU0002	KX063759.1	98.347	242	2	2	1	241	3.58E-114	424
AOTU0002	KX063754.1	98.347	242	2	2	1	241	3.58E-114	424
AOTU0002	KX063769.1	97.934	242	2	2	1	241	1.66E-112	418
AOTU0002	KX063766.1	97.934	242	2	2	1	241	1.66E-112	418
AOTU0002	KX063765.1	97.934	242	2	2	1	241	1.66E-112	418
AOTU0002	AB903025.1	97.531	243	2	1	2101	2342	2.15E-111	414
AOTU0002	GU117577.1	97.531	243	2	1	2140	2381	2.15E-111	414
AOTU0002	KX063772.1	97.521	242	2	2	1	241	7.75E-111	412
AOTU0002	KX063763.1	97.521	242	2	2	1	241	7.75E-111	412
AOTU0002	KX063768.1	97.5	240	2	2	1	239	1.00E-109	409
AOTU0004	MK262787.1	100	242	0	1	2539	2780	2.12E-121	448
AOTU0004	MK262786.1	100	242	0	1	2538	2779	2.12E-121	448
AOTU0004	MK262785.1	100	242	0	1	2494	2735	2.12E-121	448
AOTU0004	MK262783.1	100	242	0	1	2537	2778	2.12E-121	448
AOTU0004	AB903011.1	100	242	0	1	2545	2786	2.12E-121	448
AOTU0004	MW077575.1	100	242	0	1	623	864	2.12E-121	448
AOTU0004	MW077572.1	100	242	0	1	435	676	2.12E-121	448
AOTU0004	MW077560.1	100	242	0	1	480	721	2.12E-121	448
AOTU0004	MW077557.1	100	242	0	1	402	643	2.12E-121	448
AOTU0004	MW077549.1	100	242	0	1	401	642	2.12E-121	448
AOTU0005	MH619577.1	96	75	1	1	333	407	5.26E-23	121
AOTU0005	LC309538.1	96	75	1	1	1	75	5.26E-23	121
AOTU0005	MF484434.1	97.183	71	1	1	30	99	1.89E-22	119
AOTU0005	MF483882.1	98.507	67	0	1	30	96	1.89E-22	119

AOTU0005	KX220958.1	97.183	71	1	1	299	230	1.89E-22	119
AOTU0005	KY873618.1	97.183	71	1	1	44	113	1.89E-22	119
AOTU0005	KX195792.1	98.507	67	0	1	54	120	1.89E-22	119
AOTU0005	MW471062.1	97.183	71	1	1	2118	2187	1.89E-22	119
AOTU0005	MT735194.1	98.507	67	0	1	2109	2175	1.89E-22	119
AOTU0005	AJ749619.1	97.183	71	1	1	362	431	1.89E-22	119
AOTU0006	KX063721.1	100	233	0	10	1	233	2.14E-116	431
AOTU0006	KX063717.1	99.142	233	0	10	1	233	4.63E-113	420
AOTU0006	KX063742.1	98.712	233	0	10	1	233	2.15E-111	414
AOTU0006	KX063738.1	98.718	234	1	9	1	233	2.15E-111	414
AOTU0006	KX063716.1	98.712	233	0	10	1	233	2.15E-111	414
AOTU0006	KX063734.1	98.696	230	0	10	1	230	1.00E-109	409
AOTU0006	KX063736.1	97.863	234	0	9	1	234	1.30E-108	405
AOTU0006	KX063741.1	97.863	234	1	9	1	233	4.66E-108	403
AOTU0006	KX063739.1	97.863	234	1	9	1	233	4.66E-108	403
AOTU0006	KX063740.1	97.436	234	1	9	1	233	2.17E-106	398
AOTU0009	MF678005.1	92.222	90	2	1	2080	2167	1.13E-24	126
AOTU0009	XM_043070485.1	92.222	90	2	1	160	247	1.13E-24	126
AOTU0009	JX839532.1	92.222	90	2	1	339	426	1.13E-24	126
AOTU0009	U66954.2	92.222	90	2	1	309	396	1.13E-24	126
AOTU0009	MH703776.1	100	67	0	1	2101	2167	4.06E-24	124
AOTU0009	MH703756.1	100	67	0	1	2101	2167	4.06E-24	124
AOTU0009	MF484011.1	100	67	0	1	30	96	4.06E-24	124
AOTU0009	KY806552.1	100	67	0	1	40	106	4.06E-24	124
AOTU0009	KX195792.1	98.571	70	0	1	54	123	4.06E-24	124
AOTU0009	MW471062.1	100	66	0	1	2118	2183	1.46E-23	122
AOTU0015	AB902981.1	92.823	209	6	1	2244	2451	2.93E-75	294
AOTU0015	AB903004.1	92.344	209	6	1	2227	2434	3.79E-74	291
AOTU0015	LC648244.1	91.866	209	4	1	2280	2488	1.76E-72	285
AOTU0015	MF803746.1	82.381	210	9	1	598	803	1.43E-38	172
AOTU0015	MF803743.1	82.381	210	9	1	2293	2498	1.43E-38	172
AOTU0015	MF482043.1	94.118	68	2	1	30	96	1.90E-17	102
AOTU0015	LC439250.1	92.857	70	2	1	54	122	6.85E-17	100
AOTU0015	MF484969.1	92.857	70	2	1	30	98	6.85E-17	100
AOTU0015	MN904029.1	98.214	56	0	1	1	56	2.46E-16	99
AOTU0015	MT885332.1	98.214	56	0	1	2287	2342	2.46E-16	99
AOTU0020	KX063716.1	94.444	234	1	10	1	234	1.02E-94	359
AOTU0020	KX063734.1	94.017	234	1	10	1	234	4.76E-93	353

AOTU0020	KX063717.1	94.017	234	1	10	1	234	4.76E-93	353
AOTU0020	KX063741.1	93.617	235	2	9	1	234	6.16E-92	350
AOTU0020	KX063739.1	93.617	235	2	9	1	234	6.16E-92	350
AOTU0020	KX063736.1	93.617	235	1	9	1	235	6.16E-92	350
AOTU0020	KX063737.1	93.59	234	1	10	1	234	2.22E-91	348
AOTU0020	KX063740.1	93.191	235	2	9	1	234	2.87E-90	344
AOTU0020	KX063732.1	93.162	234	1	10	1	234	1.03E-89	342
AOTU0020	KX063721.1	93.162	234	1	10	1	234	1.03E-89	342
AOTU0022	LC381739.1	95.455	242	0	1	54	295	4.70E-103	387
AOTU0022	AB903002.1	86.531	245	8	1	2110	2350	2.97E-65	261
AOTU0022	AB902997.1	86.831	243	8	1	2085	2319	2.97E-65	261
AOTU0022	AB903006.1	86.42	243	8	1	2111	2345	3.84E-64	257
AOTU0022	AB903019.1	86.122	245	8	1	2102	2342	1.38E-63	255
AOTU0022	AB902999.1	86.42	243	8	1	2153	2387	1.38E-63	255
AOTU0022	AB903003.1	86.008	243	6	1	2120	2358	4.97E-63	254
AOTU0022	AB903005.1	86.008	243	8	1	2106	2340	1.79E-62	252
AOTU0022	AB902995.1	85.306	245	8	1	2111	2351	8.31E-61	246
AOTU0022	AB902969.1	85.02	247	10	1	2107	2347	2.99E-60	244
AOTU0025	MH703776.1	100	67	0	1	2101	2167	4.06E-24	124
AOTU0025	MH703756.1	100	67	0	1	2101	2167	4.06E-24	124
AOTU0025	MF484011.1	100	67	0	1	30	96	4.06E-24	124
AOTU0025	KY806552.1	100	67	0	1	40	106	4.06E-24	124
AOTU0025	KX195792.1	98.571	70	0	1	54	123	4.06E-24	124
AOTU0025	MF484434.1	100	66	0	1	30	95	1.46E-23	122
AOTU0025	MF483882.1	100	66	0	1	30	95	1.46E-23	122
AOTU0025	KX220958.1	100	66	0	1	299	234	1.46E-23	122
AOTU0025	MW471062.1	100	66	0	1	2118	2183	1.46E-23	122
AOTU0025	MT735194.1	100	66	0	1	2109	2174	1.46E-23	122
BOTU0001	AB464935.2	98.419	253	0	1	498	750	8.04E-121	446
BOTU0001	AJ867707.1	98.419	253	0	1	16	268	8.04E-121	446
BOTU0001	MN880338.1	98.024	253	0	1	1	253	3.74E-119	440
BOTU0001	MG569740.1	98.024	253	0	1	1	253	3.74E-119	440
BOTU0001	HQ622724.1	98.024	253	0	1	518	770	3.74E-119	440
BOTU0001	LC030259.1	98.008	251	0	1	172	422	4.84E-118	436
BOTU0001	AB991101.1	97.628	253	0	1	498	750	1.74E-117	435
BOTU0001	HQ595189.1	97.628	253	0	1	518	770	1.74E-117	435
BOTU0001	MK728214.1	97.233	253	0	1	1	253	8.10E-116	429
BOTU0001	KU331700.1	97.233	253	0	1	1	253	8.10E-116	429

BOTU0002	MN880352.1	98.419	253	0	1	1	253	8.04E-121	446
BOTU0002	MG569743.1	98.419	253	0	1	1	253	8.04E-121	446
BOTU0002	KX119618.1	98.419	253	0	1	421	673	8.04E-121	446
BOTU0002	KC286823.1	98.419	253	0	1	514	766	8.04E-121	446
BOTU0002	KC011109.1	98.419	253	0	1	511	763	8.04E-121	446
BOTU0002	JF420615.1	98.419	253	0	1	511	763	8.04E-121	446
BOTU0002	HQ327196.1	98.419	253	0	1	511	763	8.04E-121	446
BOTU0002	GU246825.1	98.419	253	0	1	459	711	8.04E-121	446
BOTU0002	GU246753.1	98.419	253	0	1	457	709	8.04E-121	446
BOTU0002	GU246747.1	98.419	253	0	1	456	708	8.04E-121	446
BOTU0003	KX706307.1	98.814	253	0	1	1	253	1.73E-122	451
BOTU0003	KU221939.1	98.814	253	0	1	1	253	1.73E-122	451
BOTU0003	AB991131.1	98.814	253	0	1	506	758	1.73E-122	451
BOTU0003	AB991102.1	98.814	253	0	1	504	756	1.73E-122	451
BOTU0003	AB991100.1	98.814	253	0	1	506	758	1.73E-122	451
BOTU0003	AB991097.1	98.814	253	0	1	506	758	1.73E-122	451
BOTU0003	AB991095.1	98.814	253	0	1	506	758	1.73E-122	451
BOTU0003	AB991089.1	98.814	253	0	1	504	756	1.73E-122	451
BOTU0003	AB991088.1	98.814	253	0	1	506	758	1.73E-122	451
BOTU0003	AB991025.1	98.814	253	0	1	506	758	1.73E-122	451
BOTU0004	MT585913.1	100	253	0	1	491	743	1.72E-127	468
BOTU0004	MT585911.1	100	253	0	1	492	744	1.72E-127	468
BOTU0004	MT067339.1	100	253	0	1	20	272	1.72E-127	468
BOTU0004	MN880326.1	100	253	0	1	1	253	1.72E-127	468
BOTU0004	OL636288.1	100	253	0	1	462	714	1.72E-127	468
BOTU0004	OK071780.1	100	253	0	1	20	272	1.72E-127	468
BOTU0004	LC603634.1	100	253	0	1	1	253	1.72E-127	468
BOTU0004	MZ569858.1	100	253	0	1	1	253	1.72E-127	468
BOTU0004	MW293984.1	100	253	0	1	436	688	1.72E-127	468
BOTU0004	MW237327.1	100	253	0	1	177	429	1.72E-127	468
BOTU0005	LN540363.1	100	253	0	1	177	429	1.72E-127	468
BOTU0005	KU482809.1	99.605	253	0	1	1	253	7.99E-126	462
BOTU0005	KU482650.1	99.605	253	0	1	1	253	7.99E-126	462
BOTU0005	KU482383.1	99.605	253	0	1	1	253	7.99E-126	462
BOTU0005	KU482264.1	99.605	253	0	1	1	253	7.99E-126	462
BOTU0005	KU481123.1	99.605	253	0	1	1	253	7.99E-126	462
BOTU0005	KU480604.1	99.605	253	0	1	1	253	7.99E-126	462
BOTU0005	KU480547.1	99.605	253	0	1	1	253	7.99E-126	462

BOTU0005	KU479895.1	99.605	253	0	1	1	253	7.99E-126	462
BOTU0005	KU479502.1	99.605	253	0	1	1	253	7.99E-126	462
BOTU0006	AB989911.1	98.419	253	0	1	452	704	8.04E-121	446
BOTU0006	AB991019.1	98.024	253	0	1	452	704	3.74E-119	440
BOTU0006	LC300917.1	95.652	253	0	1	1	253	3.80E-109	407
BOTU0006	KP922128.1	95.652	253	0	1	1	253	3.80E-109	407
BOTU0006	KP911486.1	95.652	253	0	1	1	253	3.80E-109	407
BOTU0006	LC306557.1	95.276	254	2	1	1	253	1.77E-107	401
BOTU0006	KP913449.1	95.257	253	0	1	1	253	1.77E-107	401
BOTU0006	JQ385261.2	95.257	253	0	1	472	724	1.77E-107	401
BOTU0006	KU106182.1	94.862	253	0	1	1	253	8.22E-106	396
BOTU0006	OK039062.1	94.862	253	0	1	152	404	8.22E-106	396
BOTU0007	AB990655.1	99.605	253	0	1	489	741	7.99E-126	462
BOTU0007	MT586022.1	99.209	253	0	1	479	731	3.72E-124	457
BOTU0007	KX119939.1	99.6	250	0	1	451	700	3.72E-124	457
BOTU0007	KX119717.1	99.209	253	0	1	455	707	3.72E-124	457
BOTU0007	KX119659.1	99.209	253	0	1	452	704	3.72E-124	457
BOTU0007	KX035429.1	99.209	253	0	1	457	709	3.72E-124	457
BOTU0007	AB989697.1	99.209	253	0	1	483	735	3.72E-124	457
BOTU0007	KF973996.1	99.209	253	0	1	222	474	3.72E-124	457
BOTU0007	KJ606838.1	99.209	253	0	1	457	709	3.72E-124	457
BOTU0007	MW507694.1	99.209	253	0	1	487	739	3.72E-124	457
BOTU0008	AB991196.1	98.419	253	0	1	505	757	8.04E-121	446
BOTU0008	AB991193.1	98.419	253	0	1	506	758	8.04E-121	446
BOTU0008	AB991144.1	98.419	253	0	1	506	758	8.04E-121	446
BOTU0008	AB991137.1	98.419	253	0	1	505	757	8.04E-121	446
BOTU0008	AB990568.1	98.419	253	0	1	505	757	8.04E-121	446
BOTU0008	AB990519.1	98.419	253	0	1	505	757	8.04E-121	446
BOTU0008	AB990494.1	98.419	253	0	1	505	757	8.04E-121	446
BOTU0008	AB990346.1	98.419	253	0	1	505	757	8.04E-121	446
BOTU0008	AB990031.1	98.419	253	0	1	506	758	8.04E-121	446
BOTU0008	KX706307.1	98.024	253	0	1	1	253	3.74E-119	440
BOTU0009	KM877155.1	98.814	253	0	1	519	771	1.73E-122	451
BOTU0009	MT585831.1	98.419	253	0	1	474	726	8.04E-121	446
BOTU0009	MK453070.1	98.419	253	0	1	460	712	8.04E-121	446
BOTU0009	MF364971.1	98.419	253	0	1	1	253	8.04E-121	446
BOTU0009	KU477721.1	98.419	253	0	1	1	253	8.04E-121	446
BOTU0009	KU464169.1	98.419	253	0	1	1	253	8.04E-121	446

BOTU0009	KU415282.1	98.419	253	0	1	1	253	8.04E-121	446
BOTU0009	KU392778.1	98.419	253	0	1	1	253	8.04E-121	446
BOTU0009	OL952610.1	98.419	253	0	1	500	752	8.04E-121	446
BOTU0009	MW927189.1	98.419	253	0	1	497	749	8.04E-121	446
BOTU0010	MT585917.1	98.419	253	0	1	507	759	8.04E-121	446
BOTU0010	MN880347.1	98.419	253	0	1	1	253	8.04E-121	446
BOTU0010	AB833681.1	98.419	253	0	1	152	404	8.04E-121	446
BOTU0010	AB833601.1	98.419	253	0	1	138	390	8.04E-121	446
BOTU0010	AB833538.1	98.419	253	0	1	137	389	8.04E-121	446
BOTU0010	AB833506.1	98.419	253	0	1	111	363	8.04E-121	446
BOTU0010	MN567150.1	98.419	253	0	1	527	779	8.04E-121	446
BOTU0010	KY908181.1	98.419	253	0	1	149	401	8.04E-121	446
BOTU0010	ON229275.1	98.419	253	0	1	1	253	8.04E-121	446
BOTU0010	MW507685.1	98.419	253	0	1	498	750	8.04E-121	446
FOTU0001	MH450386.1	100	50	0	1	1	50	1.15E-14	93.5
FOTU0001	MF484055.1	100	50	0	1	30	79	1.15E-14	93.5
FOTU0001	MF483437.1	95	60	1	1	30	88	1.15E-14	93.5
FOTU0001	MF483413.1	95	60	1	1	30	88	1.15E-14	93.5
FOTU0001	MF483277.1	95	60	1	1	30	88	1.15E-14	93.5
FOTU0001	MF482649.1	100	50	0	1	30	79	1.15E-14	93.5
FOTU0001	MF570624.1	100	50	0	1	71	120	1.15E-14	93.5
FOTU0001	OU941261.1	95	60	1	1	279	337	1.15E-14	93.5
FOTU0001	LR602161.1	100	50	0	1	100	149	1.15E-14	93.5
FOTU0001	OU973773.1	100	49	0	1	79	127	4.12E-14	91.6
FOTU0002	MN128417.1	100	242	0	1	270	511	2.12E-121	448
FOTU0002	MF615087.1	100	242	0	1	332	573	2.12E-121	448
FOTU0002	KR698883.1	100	242	0	1	269	510	2.12E-121	448
FOTU0002	KR698875.1	100	242	0	1	246	487	2.12E-121	448
FOTU0002	LC125345.1	100	242	0	1	273	514	2.12E-121	448
FOTU0002	LC125335.1	100	242	0	1	274	515	2.12E-121	448
FOTU0002	LC125291.1	100	242	0	1	278	519	2.12E-121	448
FOTU0002	KC333169.1	100	242	0	1	283	524	2.12E-121	448
FOTU0002	KC588724.1	100	242	0	1	72	313	2.12E-121	448
FOTU0002	MW238147.1	100	242	0	1	55	296	2.12E-121	448
FOTU0003	KC455921.1	97.934	242	0	1	219	460	4.63E-113	420
FOTU0003	MF615028.1	93.852	244	1	1	236	479	6.12E-97	366
FOTU0003	MT473721.1	93.443	244	1	1	294	537	2.85E-95	361
FOTU0003	MT473720.1	93.443	244	1	1	295	538	2.85E-95	361

FOTU0003	MT473719.1	93.443	244	1	1	295	538	2.85E-95	361
FOTU0003	MT473716.1	93.443	244	1	1	295	538	2.85E-95	361
FOTU0003	MT473715.1	93.443	244	1	1	293	536	2.85E-95	361
FOTU0003	MF614973.1	93.534	232	0	1	228	459	2.22E-91	348
FOTU0003	KX214375.1	92.766	235	2	1	262	495	1.33E-88	339
FOTU0003	JN400812.1	91.429	245	3	1	281	524	6.20E-87	333
FOTU0005	MT473717.1	100	242	0	1	286	527	2.12E-121	448
FOTU0005	MT473713.1	100	242	0	1	285	526	2.12E-121	448
FOTU0005	MK045389.1	100	242	0	1	257	498	2.12E-121	448
FOTU0005	MT473721.1	96.721	244	2	1	294	536	4.66E-108	403
FOTU0005	MT473720.1	96.721	244	2	1	295	537	4.66E-108	403
FOTU0005	MT473719.1	96.721	244	2	1	295	537	4.66E-108	403
FOTU0005	MT473716.1	96.721	244	2	1	295	537	4.66E-108	403
FOTU0005	MT473715.1	96.721	244	2	1	293	535	4.66E-108	403
FOTU0005	MF615028.1	93.852	244	2	1	236	478	2.20E-96	364
FOTU0005	JN400812.1	93.004	243	1	1	281	523	4.76E-93	353
FOTU0006	KX214375.1	89.344	244	2	1	262	505	1.35E-78	305
FOTU0006	JN400812.1	89.344	244	2	1	281	524	1.35E-78	305
FOTU0006	MF614973.1	90.086	232	2	1	228	459	1.75E-77	302
FOTU0006	JQ768935.1	88.934	244	2	1	281	524	6.29E-77	300
FOTU0006	LS450289.1	88.477	243	6	1	1	237	4.90E-73	287
FOTU0006	MF615028.1	87.805	246	5	1	236	479	6.34E-72	283
FOTU0006	MT473717.1	87.654	243	1	1	286	528	2.28E-71	281
FOTU0006	MT473713.1	87.654	243	1	1	285	527	2.28E-71	281
FOTU0006	MK045389.1	87.654	243	1	1	257	499	2.28E-71	281
FOTU0006	KP889680.1	88.066	243	6	1	329	565	2.28E-71	281
FOTU0007	ON352588.1	100	242	0	1	256	497	2.12E-121	448
FOTU0007	ON332098.1	100	242	0	1	316	557	2.12E-121	448
FOTU0007	OV986050.1	100	242	0	1	1	242	2.12E-121	448
FOTU0007	ON122592.1	100	242	0	1	1	242	2.12E-121	448
FOTU0007	ON130662.1	100	242	0	1	211	452	2.12E-121	448
FOTU0007	ON127895.1	100	242	0	1	263	504	2.12E-121	448
FOTU0007	ON127865.1	100	242	0	1	262	503	2.12E-121	448
FOTU0007	OM975636.1	100	242	0	1	265	506	2.12E-121	448
FOTU0007	OM975632.1	100	242	0	1	204	445	2.12E-121	448
FOTU0007	OM975597.1	100	242	0	1	259	500	2.12E-121	448
FOTU0008	MN622703.1	100	242	0	1	274	515	2.12E-121	448
FOTU0008	MN622702.1	100	242	0	1	274	515	2.12E-121	448

FOTU0008	MN622701.1	100	242	0	1	320	561	2.12E-121	448
FOTU0008	MN622700.1	100	242	0	1	274	515	2.12E-121	448
FOTU0008	MN622699.1	100	242	0	1	274	515	2.12E-121	448
FOTU0008	MN622697.1	100	242	0	1	291	532	2.12E-121	448
FOTU0008	MN622696.1	100	242	0	1	291	532	2.12E-121	448
FOTU0008	MN622695.1	100	242	0	1	292	533	2.12E-121	448
FOTU0008	MN622694.1	100	242	0	1	293	534	2.12E-121	448
FOTU0008	MN622693.1	100	242	0	1	294	535	2.12E-121	448
FOTU0009	LC599387.1	99.174	242	0	1	3288	3529	4.60E-118	436
FOTU0009	LC599386.1	99.174	242	0	1	3604	3845	4.60E-118	436
FOTU0009	MW161066.1	99.174	242	0	1	312	553	4.60E-118	436
FOTU0009	MW161065.1	99.174	242	0	1	312	553	4.60E-118	436
FOTU0009	AB474395.1	97.881	236	0	1	334	569	2.79E-110	411
FOTU0009	KR266147.1	91.556	225	3	1	1	222	3.76E-79	307
FOTU0009	KF617467.1	90.598	234	8	1	309	535	3.76E-79	307
FOTU0009	MT386399.1	81.935	155	6	1	337	487	1.13E-24	126
FOTU0009	MK991847.1	87.069	116	6	1	148	260	1.13E-24	126
FOTU0009	MT095914.1	77.35	234	14	1	1	226	1.46E-23	122
FOTU0010	LC514957.1	96.694	242	1	1	287	525	6.03E-107	399
FOTU0010	LC514929.1	96.694	242	1	1	290	528	6.03E-107	399
FOTU0010	LC514927.1	96.694	242	1	1	290	528	6.03E-107	399
FOTU0010	MW561274.1	96.694	242	1	1	311	549	6.03E-107	399
FOTU0010	LC514967.1	93.574	249	6	1	297	542	7.91E-96	363
FOTU0010	LC514966.1	93.574	249	6	1	297	542	7.91E-96	363
FOTU0010	LC514965.1	93.574	249	6	1	295	540	7.91E-96	363
FOTU0010	LC514962.1	93.574	249	6	1	289	534	7.91E-96	363
FOTU0010	LC514961.1	93.574	249	6	1	296	541	7.91E-96	363
FOTU0010	LC514959.1	93.574	249	6	1	297	542	7.91E-96	363
FOTU0011	AB474394.1	96.281	242	0	1	317	558	2.17E-106	398
FOTU0011	KU062657.1	91.02	245	6	1	20	261	1.04E-84	326
FOTU0011	KU188573.1	91.915	235	3	1	19	251	1.04E-84	326
FOTU0011	MN626358.1	90.947	243	4	1	251	490	3.73E-84	324
FOTU0011	MN983247.1	90.947	243	4	1	274	513	3.73E-84	324
FOTU0011	MN983243.1	90.947	243	4	1	274	513	3.73E-84	324
FOTU0011	MN983242.1	90.947	243	4	1	275	514	3.73E-84	324
FOTU0011	KU559652.1	90.947	243	4	1	177	416	3.73E-84	324
FOTU0011	MW019433.1	90.909	242	3	1	298	536	1.34E-83	322
FOTU0011	MW019430.1	90.909	242	3	1	298	536	1.34E-83	322

Supplementary Table S5 WOCNA tests and parameters used.

Terms	Abbreviation
Scale-free Topology Model Fit	R^2
Mean Connectivity Score	κ
Soft-thresholding Powers	β
Sub-network OTU Count	OTU_N
OTU Sub-network Size	Mod_{min}
Max Tree Cut Height	Mod_{maxcut}
OTU Sub-network Number	Mod_N

Test/Variable	Result/Setting
Hellinger Distance Clustering for Outliers	No outliers detected

Variable	Setting
OTU_N (Cascades)	241
R^2 (Cascades)	0.84
β (Cascades)	7
κ (Cascades)	2.1
Mod_{min} (Cascades)	14
Mod_{maxcut} (Cascades)	2.25
Mod_N (Cascades)	7
OTU_N (Rockies)	110
R^2 (Rockies)	0.81
β (Rockies)	9
κ (Rockies)	1.32
Mod_{min} (Rockies)	6
Mod_{maxcut} (Rockies)	1.75
Mod_N (Rockies)	8

Module	Stability Score (SABRE)
Turquoise (Cascades)	0.37
Blue (Cascades)	0.12
Brown (Cascades)	0.12

Yellow (Cascades)	0.11
Green (Cascades)	0.11
Black (Cascades)	0.1
Red (Cascades)	0.1
Turquoise (Rockies)	0.32
Blue (Rockies)	0.16
Brown (Rockies)	0.17
Yellow (Rockies)	0.17
Green (Rockies)	0.17
Black (Rockies)	0.17
Red (Rockies)	0.15
Pink (Rockies)	0.15

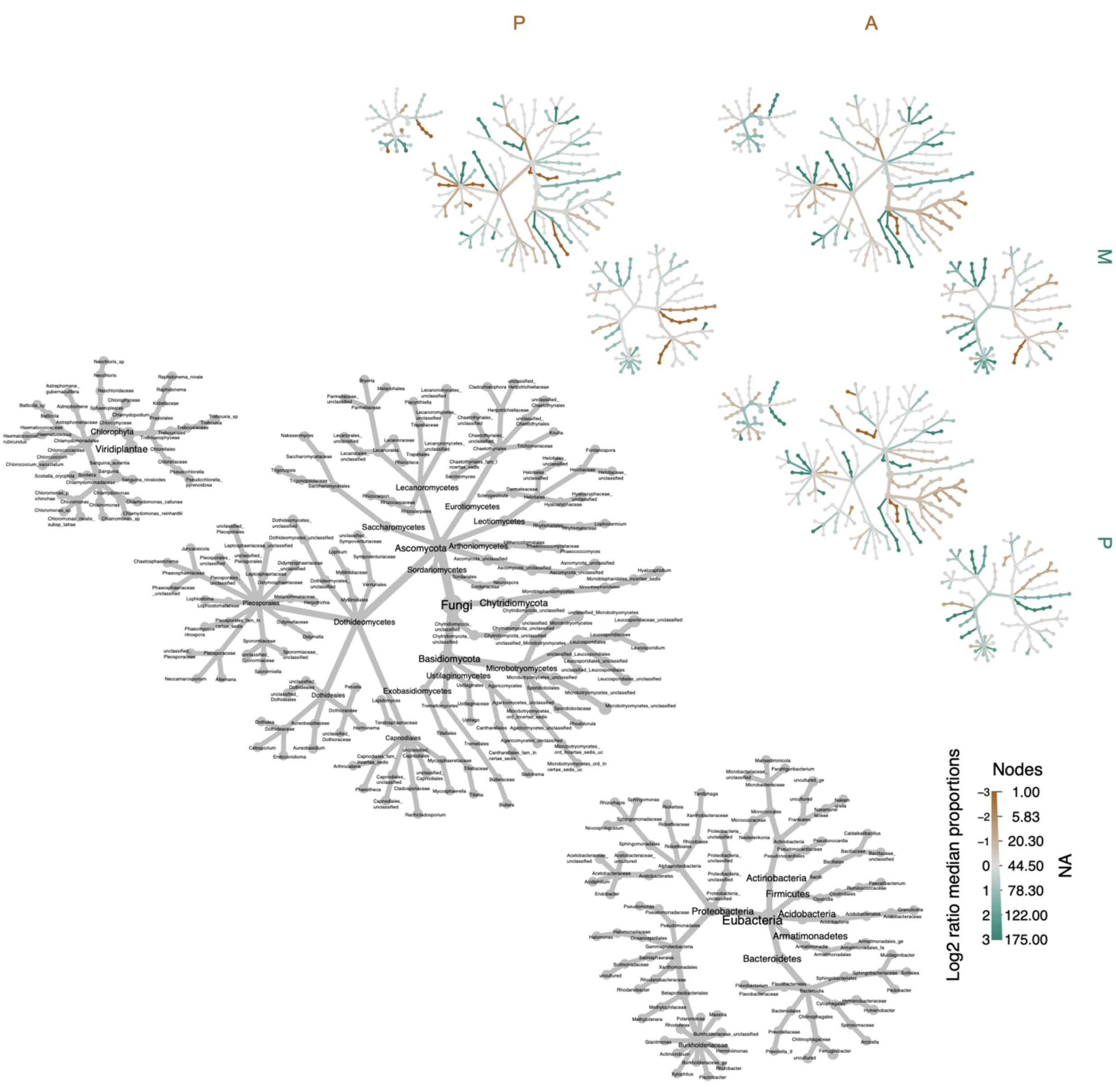
P
A
M
P
Viridiplantae
Chlorophyta
Fungi
Ascomycota
Basidiomycota
Chytridiomycota
Dothideomycetes
Sordariomycetes
Saccharomycetes
Lecanoromycetes
Eurotiomycetes
Leotiomycetes
Arthoniomycetes
Microbotryomycetes
Ustilaginomycetes
Exobasidiomycetes
Pleosporales
Dothideales
Capnodiales
Eubacteria
Proteobacteria
Actinobacteria
Firmicutes
Acidobacteria
Armatimonadetes
Bacteroidetes
Burkholderiaceae
Nodes
1.00
5.83
20.30
44.50
78.30
122.00
175.00
Log2 ratio median proportions
NA

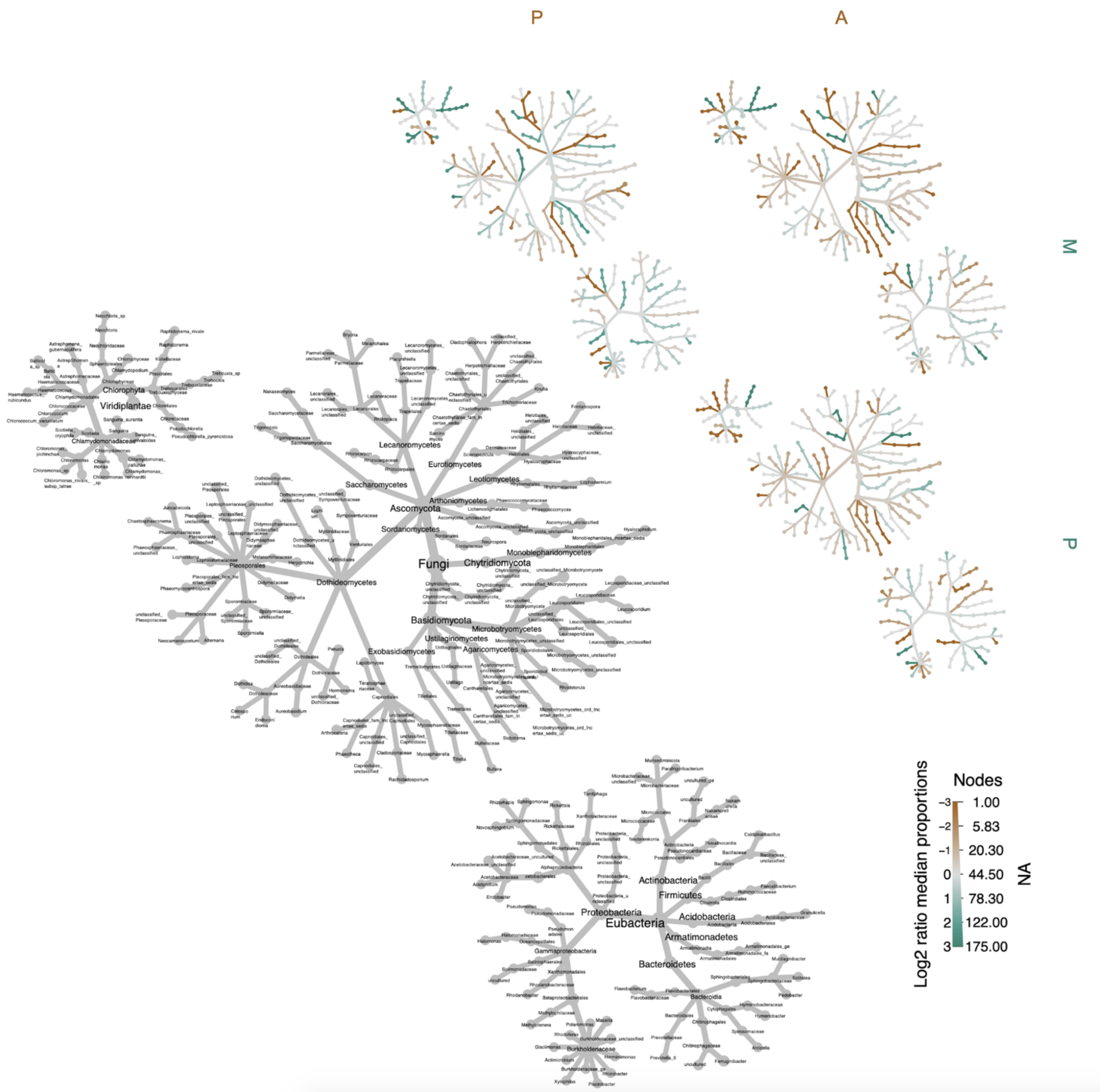

Supplementary Figure S1 (a-b) Metacoder generated pairwise heat-tree matrices comparing OTU lineages between algae (genus), bacteria (family), and fungi (family) across zones M, P and A in the (a) Cascades and (b) Rockies. Colored nodes indicate the log2-ratio of median proportions between OTU zone pairs. Green indicates higher OTU proportion for the zone listed on the vertical axis, while brown indicates a higher proportion for the horizontal axis. Gray indicates no significant difference in proportion between zone pairs. A tree legend is provided in gray to the left.

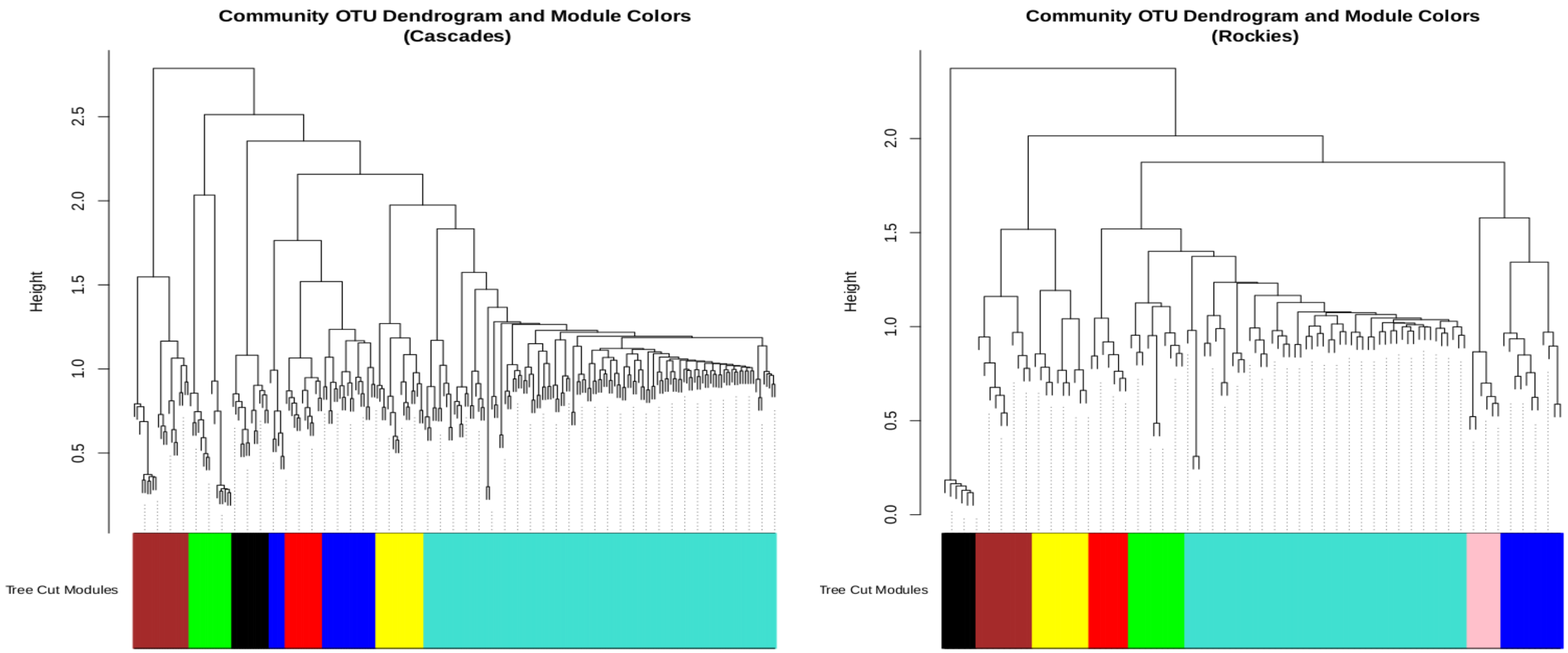

Supplementary Figure S2 Dendrogram showing hierarchical clustering of community level OTUs in the Cascade and Rocky Mountains clustered as soft-threshold TOM dissimilarity adjacencies of Hellinger distances. The colors below the dendrogram for the Cascade Mountains represent seven colorized modules based on a $Mod_{min} = 14$ and $Mod_{maxcut} = 2.25$ where module stability is: turquoise = 0.37, blue = 0.12, brown = 0.12, yellow = 0.11, green = 0.11, black = 0.10, red = 0.10. The colors below the dendrogram for the Rocky Mountains represent eight colorized modules based on a $Mod_{min} = 6$ and $Mod_{maxcut} = 1.75$ where module stability is: turquoise = 0.32, blue = 0.16, brown = 0.17, yellow = 0.17, green = 0.17, black = 0.17, red = 0.15, pink = 0.15. Note, modules with same color between regions do not necessarily represent the same subset of OTUs.

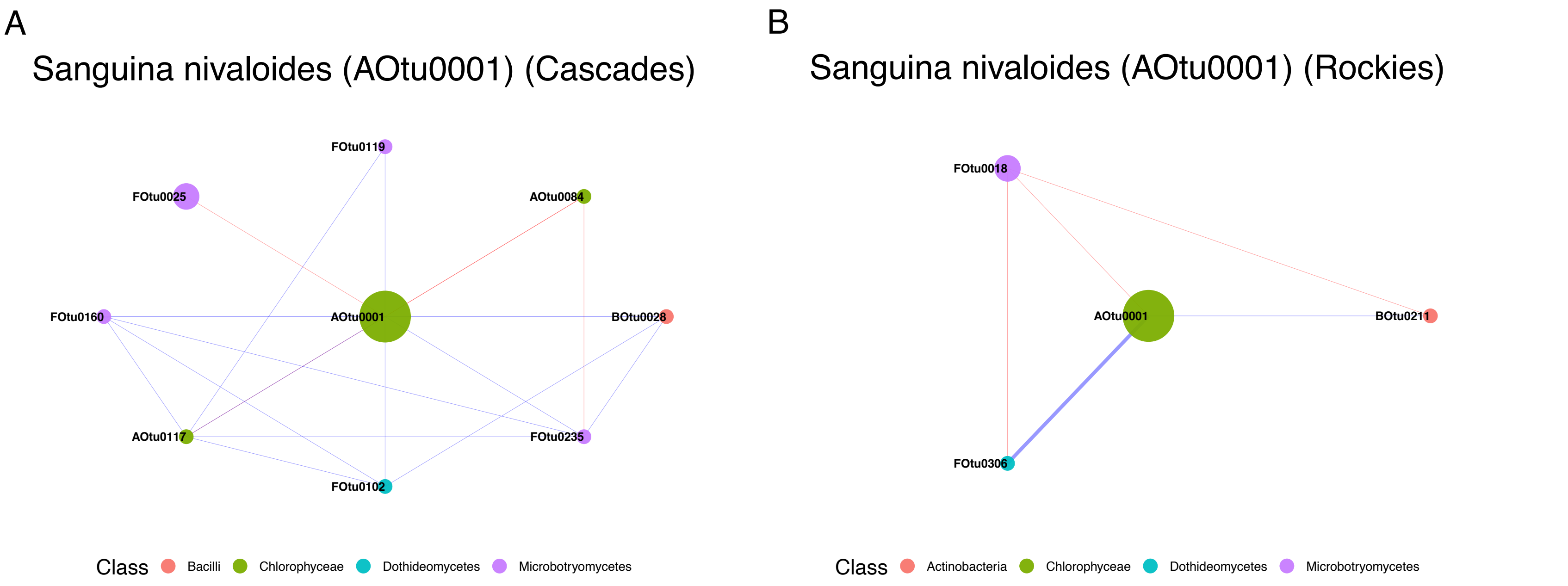

Supplementary Figure S3 (a-b) *Sanguina nivaloides* (AOtu0001) co-association network from the Cascade (Edges = 18; Vertices = 9; Transitivity = 0.81; Average-Path Length = 1.5) and (Edges = 5; Vertices = 4; Transitivity = 0.83; Average-Path Length = 1.17) Rocky Mountains. Red lines indicate a negative correlation while blue lines indicate a positive correlation. Thin lines have a $|K| < 0.6$; moderate lines have a $|K| \geq 0.6$ and < 0.8; thick lines have a $|K| \geq 0.8$. Vertex colors are randomly generated and not necessarily the same across graphics.

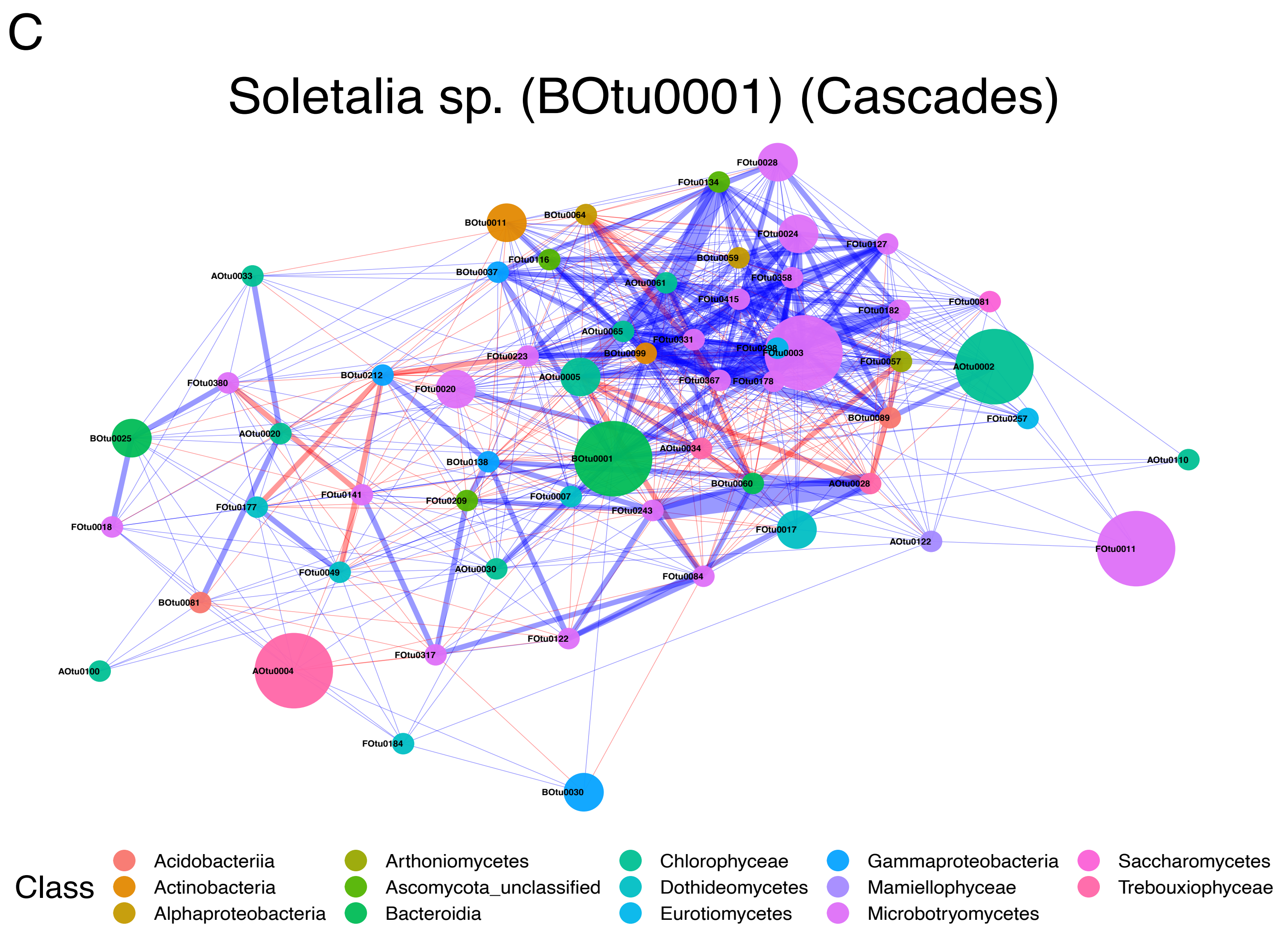

Supplementary Figure S3 (c) *Solitalea* sp. (BOtu0001) co-association network from the Cascade Mountains (Edges = 655; Vertices = 58; Transitivity = 0.68; Average-Path Length = 1.6).

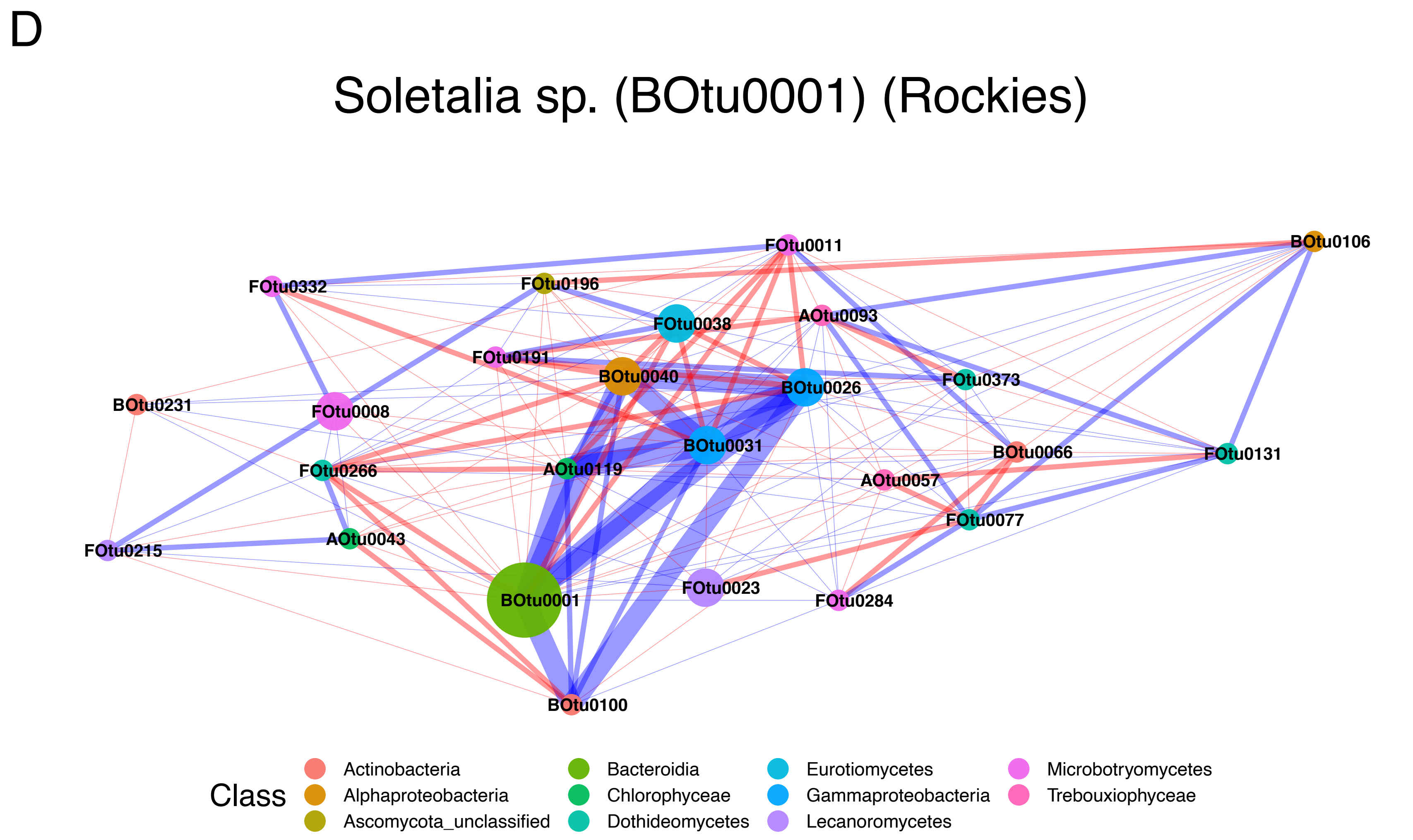

Supplementary Figure S3 (d) *Solitalea* sp. (BOtu0001) co-association network from the Rocky Mountains (Edges = 161; Vertices = 25; Transitivity = 0.72; Average-Path Length = 1.4).

E

unclassified Chytridiomycota (FOtu0001) (Cascades)

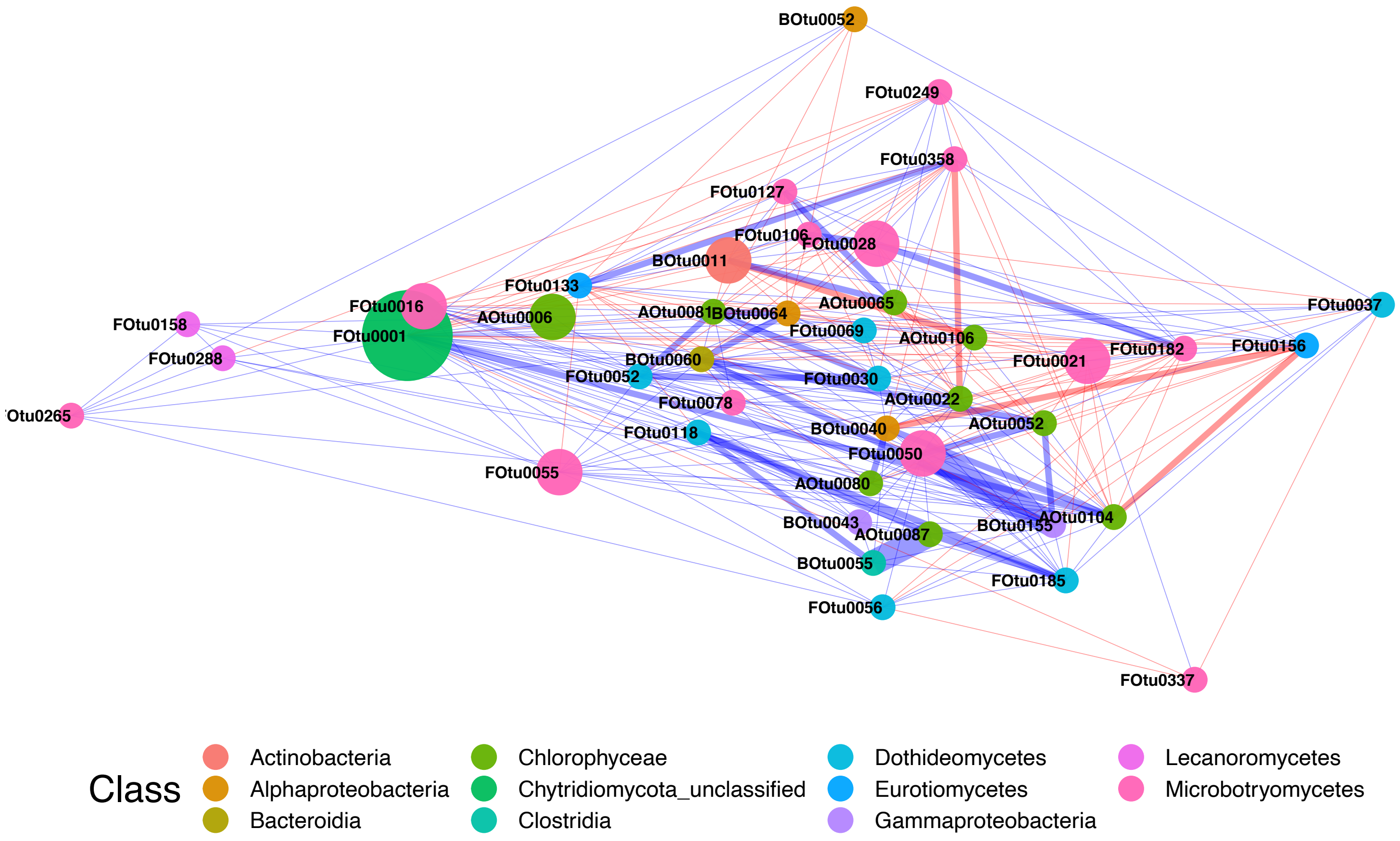

Supplementary Figure S3 (e) unclassified Chytridiomycota (FOtu0001) co-association network from the Cascade Mountains (Edges = 655; Vertices = 58; Transitivity = 0.68; Average-Path Length = 1.6).

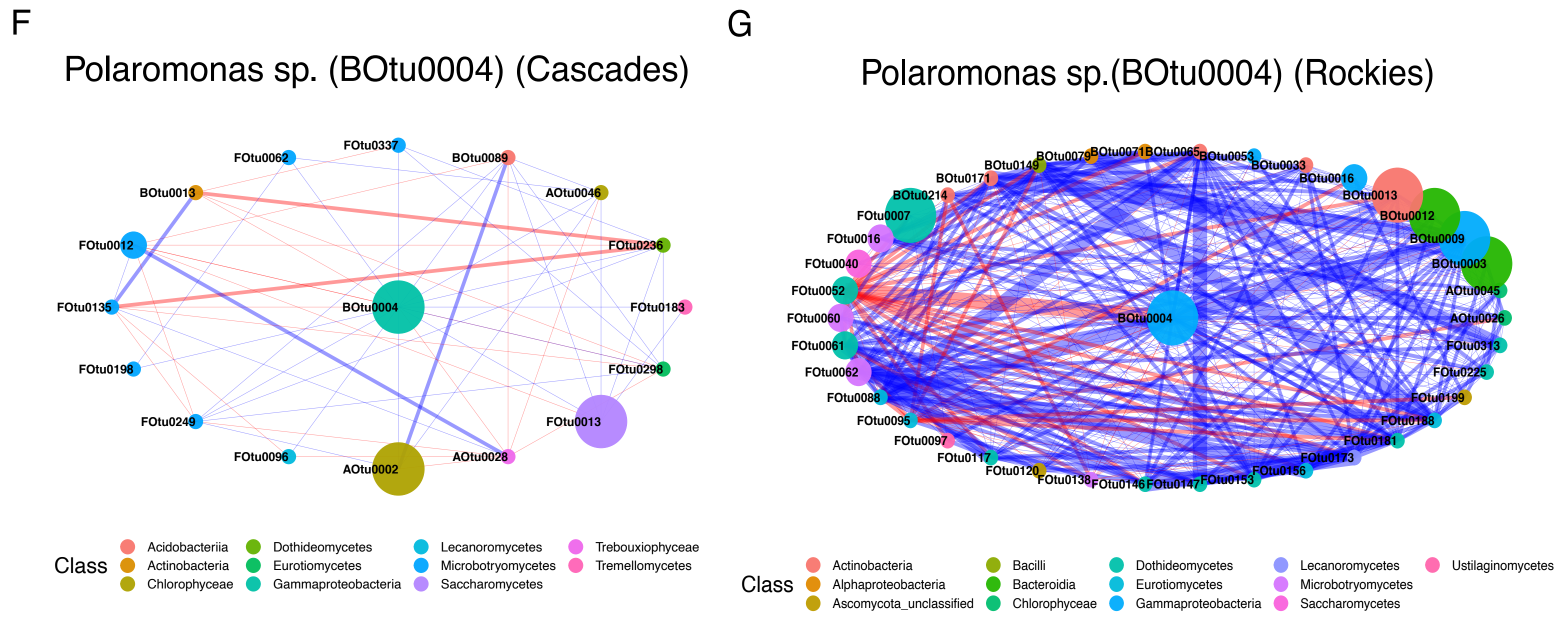

Supplementary Figure S3 (f-g) *Polaromonas* sp. co-association networks from the Cascade (Edges = 55; Vertices = 517; Transitivity = 0.75; Average-Path Length = 1.6) and Rocky Mountains (Edges = 376; Vertices = 39; Transitivity = 0.72; Average-Path Length = 1.49).

A

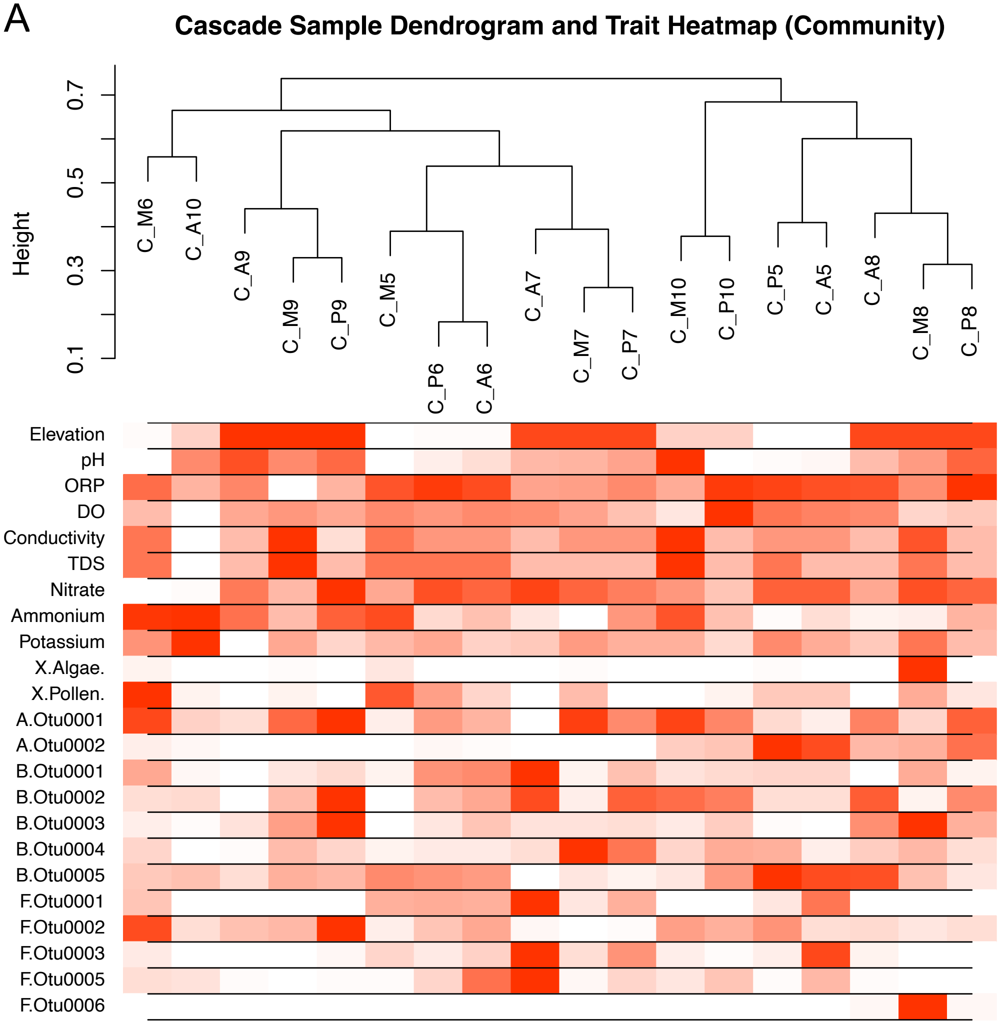
Cascade Sample Dendrogram and Trait Heatmap (Community)
Height
0.1
0.3
0.5
0.7
C_M6
C_A10
C_A9
C_M9
C_P9
C_M5
C_P6
C_A6
C_A7
C_M7
C_P7
C_M10
C_P10
C_P5
C_A5
C_A8
C_M8
C_P8
Elevation
pH
ORP
DO
Conductivity
TDS
Nitrate
Ammonium
Potassium
X.Algae.
X.Pollen.
A.Otu0001
A.Otu0002
B.Otu0001
B.Otu0002
B.Otu0003
B.Otu0004
B.Otu0005
F.Otu0001
F.Otu0002
F.Otu0003
F.Otu0005
F.Otu0006

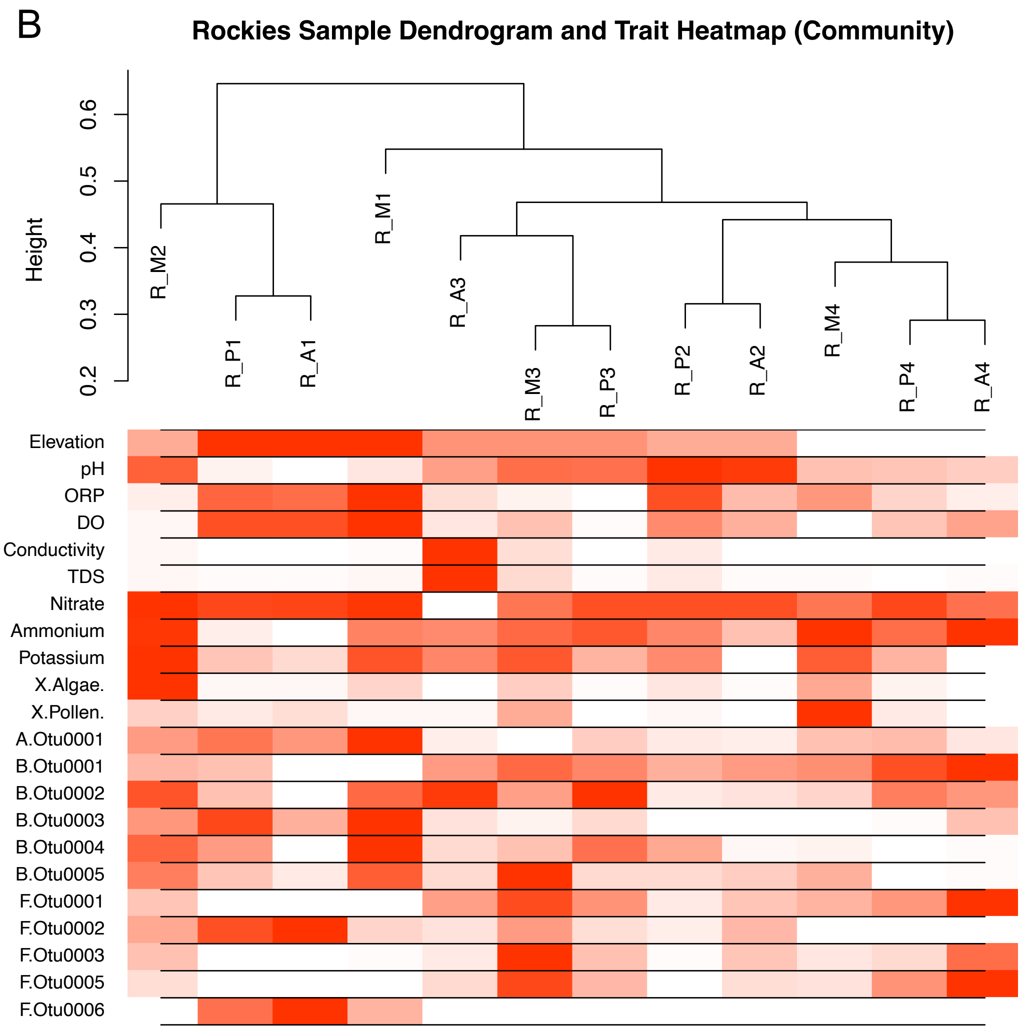

Supplementary Figure S4 (a-b) Hierarchical clustering of Cascade (a) and Rocky Mountain (b) snow community samples plotted against environmental traits and OTU relative abundances. White squares indicate a low numeric value for an environmental trait or OTU relative abundance and an increasing intensity of red indicates a higher numeric value. Sample sets were clustered to assess whether samples clustered by snow zones (M, P and A) or by bloom site for the Cascade and Rocky Mountains.

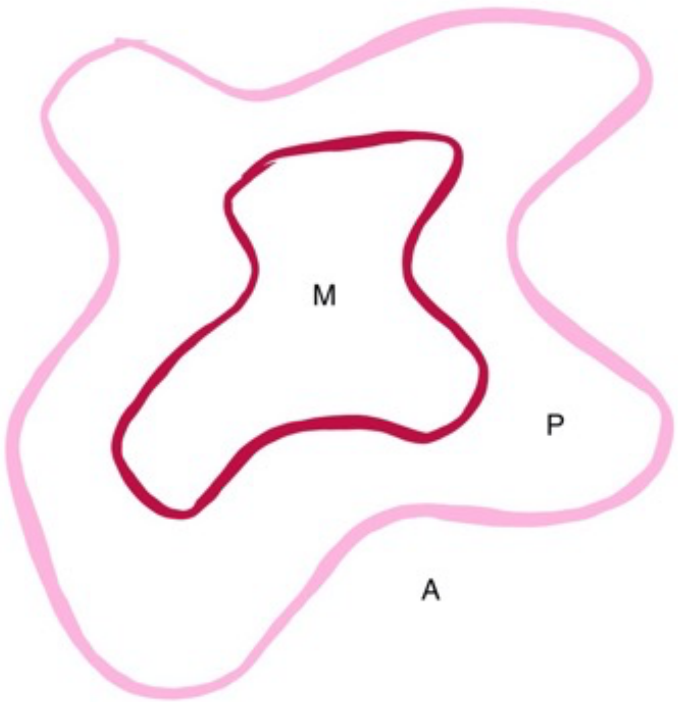

Supplementary Figure S5 Snow algae bloom zone schematic map showing medial (M), peripheral (P) and adjacent (A). Medial snows are characterized by a deep red hue and a high density of snow algae, peripheral zones by a light pink hue and medium snow algae density and adjacent zones by a white hue with low to no snow algae present.

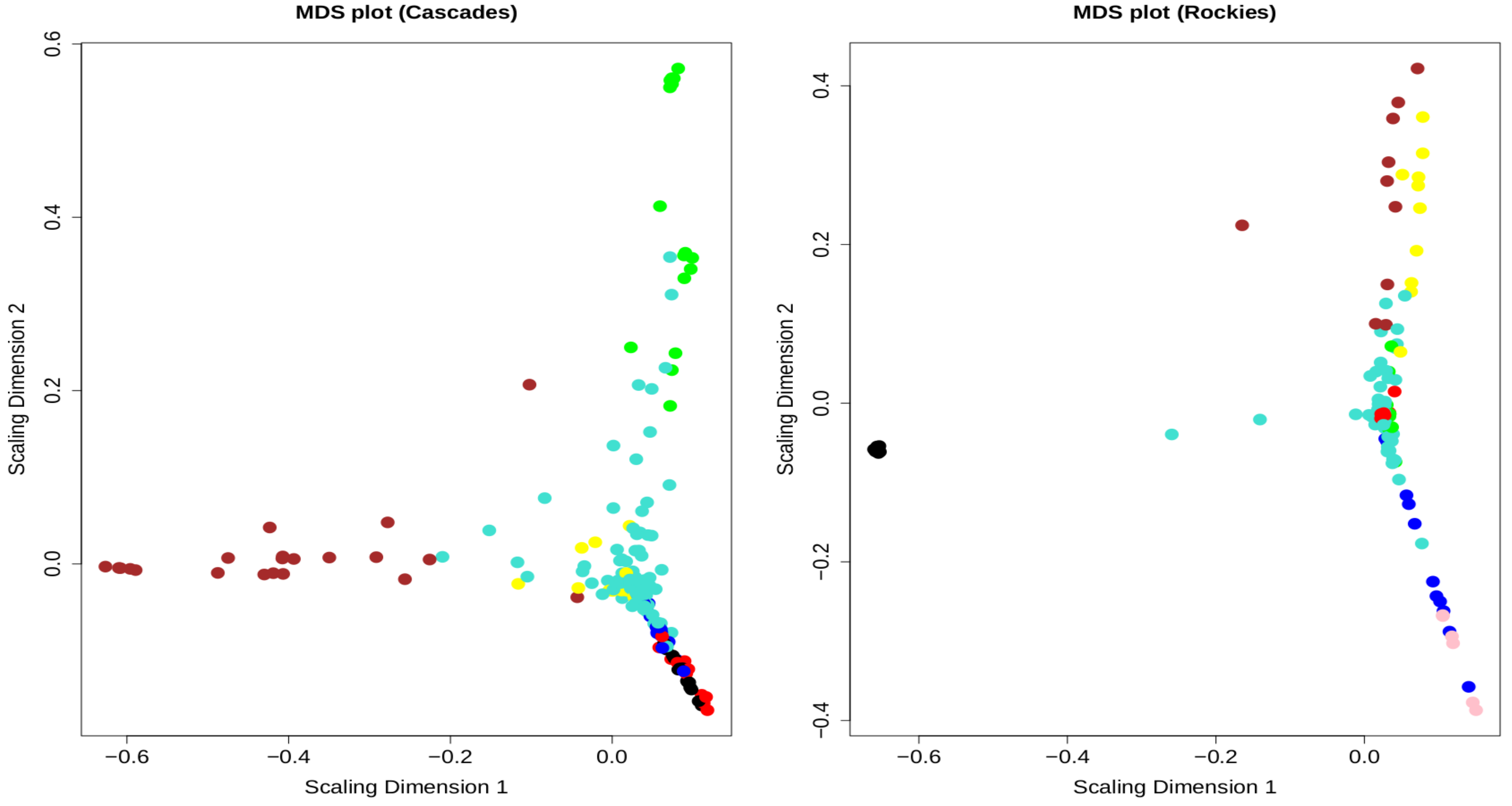

Supplementary Figure S6 MDS plots showing snow microbial community OTUs in the Cascade (seven colorized modules) and Rocky Mountains (eight colorized modules). Dimensions 1 and 2 represent the first and second principal components respectively. Modules corresponding to fingers and fingertips may suggest intramodular hubs of OTUs; this is particularly evident for green and brown modules in the Cascades and black, brown and a combination of blue/pink in the Rockies.
